# London Trauma Conference 2019: London, United Kingdom. 10-13 December 2019

**DOI:** 10.1186/s13049-020-0711-6

**Published:** 2020-03-05

## 1. Whole blood transfusion versus component therapy in adult trauma patients with acute major haemorrhage: a systematic review

### Pascale Avery^1^, Sarah Morton^2^, Harriet Tucker^3^, Laura Green^4^, Anne Weaver^5^, Ross Davenport^6^

#### ^1^North Bristol NHS Trust, Bristol, UK; ^2^Imperial College Healthcare NHS Trust, London, UK; ^3^Air Ambulance Kent Surrey Sussex, UK; ^4^Haematology Department, Barts Health NHS Trust, London, UK; ^5^London’s Air Ambulance, The Royal London Hospital, London, UK; ^6^Blizard Institute, Queen Mary University of London, London UK

##### **Correspondence:** Pascale Avery (pascale.avery@nhs.net)

**Background**

In the era of damage control resuscitation of trauma patients with acute major haemorrhage, transfusion practice has evolved to blood components administered in a fixed ratio that closely approximates whole blood (WB). However, there is a paucity of evidence supporting the optimal transfusion strategy in these patients. The primary objective was to establish if there is an improvement in survival at 30-days with the use of WB transfusion compared with components therapy (CT) in adult trauma patients with acute major haemorrhage.

**Methodology**

A systematic literature search was performed (PubMed, Web of Science, Cochrane, OVID, Embase and the Transfusion Evidence Library) to identify studies comparing WB transfusion with CT in adult trauma patients with acute major haemorrhage. Studies which did not report mortality were excluded. Methodological quality of included studies was interpreted using the Cochrane risk of bias tool, and rated using the Grading of Recommendations Assessment, Development and Evaluation (GRADE) approach.

**Results**

Search of the databases identified 1816 records, and six studies met the inclusion criteria involving 3255 patients. Of the three studies reporting 30-day mortality, one study presented a statistically significant difference between WB and CT, and two found no statistical difference. The studies reporting in-hospital mortality found no statistical difference in unadjusted mortality, but both reported statically significant logistic regression analyses demonstrating that those with a WB transfusion strategy were less likely to die.

**Conclusion**

Recognising the limitations of this systematic review relating to the nature of included trials, it does not provide evidence to support or reject use of WB transfusion compared with CT for adult trauma patients with acute major haemorrhage. Larger prospective, randomised trials are required to better understand if WB improves survival in adult trauma patients with acute major haemorrhage compared with CT.

## 2. Prophylactic antibiotics for thoracostomy in penetrating thoracic trauma - a systematic literature review

### James J Hale^1^, Andrew Saunders^2^

#### ^1^Department of Anaesthesia, Royal Infirmary of Edinburgh, Edinburgh, UK; ^2^Department of Emergency Medicine, Royal Infirmary of Edinburgh, Edinburgh, UK

##### **Correspondence:** James J Hale (james.hale@nhs.net)

**Background**

Intrathoracic infection following penetrating thoracic trauma is a significant complication. Patients with penetrating trauma often have finger or tube thoracostomies performed prehospitally or in the emergency department (ED). Prophylactic antibiotics in these patients are contentious with contradictory published studies. Guidelines reflect this controversy, without firm recommendations for or against antibiotics.

**Methodology and results**

We conducted a systematic literature review of prophylactic antibiotics in penetrating thoracic trauma requiring thoracostomy both prehospitally and in the ED.

Six meta-analysis and eleven randomised trials were identified of patients undergoing thoracostomy in the ED. Only one meta-analysis looked purely at penetrating trauma, and this was a subgroup analysis [1]. It included all relevant randomised trials identified in the literature search and showed a positive effect for antibiotics with an OR 0.28 (95% CI 0.14 – 0.57) and NNT of 4.8.

Two cohort studies were identified of patients undergoing thoracostomy prehospitally [2,3]. Both compared infection rate in a prehospital and ED group, neither showing a difference between cohorts. Both included a mix of penetrating and blunt trauma, neither gave data on antibiotic use and there were significant differences between the cohorts.

**Discussion**

Despite significant differences between penetrating and blunt trauma, the majority of trials combine both groups. The only meta-analysis of purely penetrating trauma showed a positive signal. Weaknesses include variation in antibiotic regimes and the definition of infections. Despite this, we believe there is level 2 evidence (Oxford CEBM) for the use of prophylactic antibiotics.

Administering antibiotics prehospitally requires time and personnel, it risks delaying other time-critical interventions and transfer to definitive care. In the ED, there is proven benefit to the administration of antibiotics at thoracostomy. When time permits, we would recommend the same for prehospital patients but further research is required to characterise the effect in prehospital patients.

**References**

1. Bosman A, de Jong MB, Debeij J, van den Broeck PJ, Schipper IB. Systematic review and meta-analysis of antibiotic prophylaxis to prevent infections from chest drains in blunt and penetrating thoracic injuries. Br J Surg. 2012; 99: 506-513.

2. York D, Dudek L, Larson R, Marshall W, Dries D. A comparison study of chest tube thoracostomy: Air medical crew and in-hospital trauma service. Air Med J. 1993 Jul; 12(7): 227-9

3. Spanjersberg WR, Ringburg AN, Bergs EA, Krijen P, Schipper IB. Prehospital Chest Tube Thoracostomy: Effective Treatment or Additional Trauma? Journal of Trauma. 2005 Sep; 59(3): 788-793

## 3. Iliac artery morphology: A cadaveric study on the impact of anatomical variation on resuscitative endovascular balloon occlusion of the aorta (REBOA)

### Rachel Poustie^1^, Manik Chana^2^, Paula Vickerton^1^

#### ^1^Barts and The London School of Medicine and Dentistry, Queen Mary, University of London, 4 Newark Street, Whitechapel, London, UK; ^2^The Institute of Prehospital Care, 77 Mansell Street, London, UK

##### **Correspondence:** Rachel Poustie (r.e.poustie@smd14.qmul.ac.uk)

**Background**

Non-compressible torso haemorrhage is a leading cause of mortality following trauma. These patients often exsanguinate to death prior to meaningful attempts at definitive haemostasis. Resuscitative Endovascular Balloon Occlusion of the Aorta (REBOA) is an emerging technique, which aims to limit ongoing haemorrhage and has been shown to be feasible in the prehospital phase. REBOA utilises the common femoral artery (CFA) to gain access to the aorta. The ease of access to the aorta can have a direct impact on the success of such interventions. The aim of this study is to determine if iliac tortuosity or lumen diameter influences the time taken to perform REBOA in human cadaveric model.

**Methods**

This study was carried out in twenty-six, soft-fixed embalmed cadavers (17 male; 9 female). REBOA was performed and timed from first needle puncture to inflation of balloon in the cadavers. The tortuosity index and lumen diameter of the iliac arteries was measured. Linear regression was applied to assess the relationship between aortic occlusion and vascular morphology. The length of the CFA and qualitative data around vascular access was also recorded.

**Results**

A strong positive correlation between tortuosity and time to occlusion (R^2^=0.39, p=0.002) was measured. The correlation between lumen diameter and time to occlusion was not statistically significant (R^2^=0.01, p=0.72). It was found that in cadavers with more tortuous arteries more resistance to inserting guide wires was felt. Furthermore only cadavers with a tortuosity index ≥1.35 experienced vascular damage by the REBOA procedure. There was wide variation of lengths of CFA (16 – 73mm) amongst the cadavers.

**Conclusion**

This is the first U.K. based human cadaveric REBOA study. The data suggests that having tortuous vasculature not only delays the time to complete aortic occlusion but also increases the risk of complications following REBOA.

## 4. Outcomes in Managing Open Abdomens in Emergency Surgery: 8 years’ Experience

### Silva Barbosa^1^, Aiysha Puri^2^, Eugenia Bonelli^2^, Simon McCluney^2^, Nebil Behar^2^

#### ^1^Cardioinfantil Fundacion , Bogota, Colombia; ^2^Chelsea and Westminster NHS Foundation Trust, London, UK

##### **Correspondence:** Eugenia Bonelli (Eugenia.Bonelli@chelwest.nhs.uk)

Introduction:

An open abdomen is an infrequent but essential part of a surgeon’s armamentarium when closure is not possible due to sepsis, oedema and domain loss. Failure to close early will result in slow healing laparostomy wound with morbidity and mortality. We describe our practice of closure in 30 consecutive patients. Newer techniques should be assessed against this baseline. Within a dedicated emergency surgery setting, experience accumulates over time. We sought to analyse the outcomes in these complex patients.

Methods:

In this study we describe our outcomes from a single unit, providing seventy percent of Emergency Surgery service for the hospital. We performed a retrospective analysis of a prospectively collected database over an eight year period. Electronic Patient Records were interrogated for type of closure, time to closure, length of hospital stay, morbidity and mortality in patients requiring laparostomy for a variety of reasons.

Results:

35 patients were identified, 4 patients were excluded due to incomplete data. Median age was 55 (16-89 years old), 55% of patients were male, 78% of patients were ASA 3 and above. 37% remained in ITU for more than 30 days, and the median length of stay was 49 days (12-120 days). 55% (n=15) of patients had primary fascial closure without mesh. 12 patients were closed with biological inlay mesh, 75% of mesh closures were bridged. In 4 patients, closure was not possible.

Whilst 78% of patients experienced a post-operative complication (26% wound infection), no patient required re-laparotomy necessitating disruption of final closure. There were no inpatient deaths, and 30-day mortality was 0%.

Conclusion:

Laparostomy is a challenging condition to manage, with high morbidity. Low mortality and satisfactory outcomes can be achieved with early closure using abdominal vacuum, component separation and, biological mesh bridge, if fascial approximation is not possible, in these frequently septic and very sick patients.

## 5. A simple intervention to increase VTE prophylaxis compliance in neck of femur fracture patients

### Keshav K. Gupta, Ranjodh S. Sanghera, Sreenadh Gella

#### Sandwell and West Birmingham Hospitals NHS Trust, Birmingham, UK

##### **Correspondence:** Keshav K. Gupta (keshav.gupta@nhs.net)

**Background**

Fragility and hip fractures are increasingly more common due to an ageing population. They represent a significant healthcare burden due to the high morbidity and mortality associated with each fracture (1). Venous thromboembolism (VTE) is one of the dangerous and significant complications, thus prevention is key. Thromboprophylaxis has shown to significantly reduce the incidence of fatal VTE complications.

**Methods**

We assessed compliance with VTE prophylaxis in post-operative neck of femur fracture patients. The National Institute of Health and Clinical Excellence (NICE) guidance recommends all patients with hip fractures should have 28-35 days of thromboprophylaxis starting 6-12 hours after surgery provided there are no contraindications (2).

**Results**

We analyzed 95 patient records between April and June 2018 and looked at 82 records following a strict inclusion/exclusion criteria. We found 77/82 patients were covered with anticoagulation at discharge. We set up a simple intervention with the new cycle of junior doctors by creating posters in key prescribing areas of wards to remind them of the NICE guidance. We repeated the methodology between August and October 2018 and found 64/64 patients were covered with anticoagulation at discharge.

**Conclusions**

Our findings show a simple intervention can radically improve compliance to national guidance. This is in keeping with a previous similar study conducted in 2014 where compliance to thromboprophylaxis prescribing following neck of femur fractures improved following a simple intervention of small labels on prescribing computers (3). It is difficult to accurately assess post-operative VTE incidence as these are commonly admitted under medics rather than surgeons.

**References**

1. McNamara, I., Sharma, A., Prevost, T. and Parker, M., 2009. Symptomatic venous thromboembolism following a hip fracture: incidence and risk factors in 5,300 patients. *Acta orthopaedica*, *80*(6), pp.687-692.

2. NICE. Venous thromboembolism in over 16s: reducing the risk of hospital-acquired deep vein thrombosis or pulmonary embolism. NICE guidance [NG89]. Available from https://www.nice.org.uk/guidance/ng89/chapter/Recommendations#interventions-for-people-having-orthopaedic-surgery.

3. Sinha, P., Najefi, A.A. and Hambidge, J., 2014. A simple measure to improve the rates of thromboprophylaxis prescription post surgical fixation of neck of femur fractures in a district general hospital. *BMJ Open Quality*, *3*(1), pp.u202991-w1956.

## 6. Development of web based electronic platform for road traffic injury surveillance at apex trauma centre in India - An Innovation

### Gaurav Kaushik, Amit Gupta, Kapil Dev Soni, Narendra Choudhary, Ankita Shrama

#### Division of Trauma Surgery and Critical Care, Jai Prakash Narayan Apex Trauma Centre, All India Institute of Medical Sciences, New Delhi, India

##### Gaurav Kaushik (grv_kk@yahoo.com)

**Background:** A Trauma Registry is a vital component of a trauma system [1]. Trauma registry is a user-friendly, electronic hospital-based injury surveillance tool that systematically stores data of trauma patients which can be used to accurately assess the true burden of injury [2].

**Methods:** A web based electronic platform has been designed to use in Emergency Department which can be used on android phones/laptops with internet access. It has two panels, 1) is administrative panel another one (2) is user panel. Both the panels are password protected and can be accessed by the investigator, data collectors or another authorized person. Admin panel is to create the users as per the requirement of the study site. User panel is being used for data collection by the trained data collectors.

**Results:** From Oct 1^st^ 2017 till 31^st^ May 2019, total trauma patients arrived at JPNATC were 40747. Of these, 9085 RTI patients triaged to red and green area were recruited in the registry. The data have been entered into the registry software. Of 9085 RTI patients, 29% were admitted to the hospital, 49% were discharged and 22% fall in other category (abscond, LAMA, transferred, deaths).

The data collection was carried out by the research nurses posted in the emergency department on rotation for 24/7 in shift duties i.e. 8am to 2pm, 2pm to 8pm and 8pm to 8am with one night off. The average time to enter the data electronically takes approximately 15 min and on an average 25-30 RTI patients (Yellow and Red) arrive in ED per day. All the data collectors were provided a tablet dedicated to the registry data entry.

**Conclusion:** There is no trauma registry in India so far for the road traffic injury patients. Present innovation would lay the foundation of national Trauma Registry in India.

**References**

1. Brook EM: The current and future use of registries in health information systems. In *Publication No 8* Geneva: World Health Organization; 1974.

2. Gliklich RE, Dreyer NA, Leavy MB, editors. 3rd edition. Rockville (MD): Agency for Healthcare Research and Quality (US); 2014 Apr. Report No.: 13(14).

## 7. An investigation in to the current location of HIOWAA assets

### James Wigley^**1**^**,** Vivienne Parsons^2^, Simon Hughes^1^, Charles Deakin^1^

#### ^1^Department of Anaesthetics, University Hospital Southampton, Southampton, UK; ^2^ Specialist Business Analyst Business Intelligence, South Central Ambulance NHS Foundation Trust, Oxfordshire UK

##### **Correspondence:** James Wigley (james.wigley@uhs.nhs.uk)

**Background**

Critical care provision across Hampshire and the Isle of Wight areas are currently covered by a HEMS service between the hours of 07:00 and 02:00. This service includes both helicopter and rapid response vehicle (RRV) platforms with each of those assets being based at the Thruxton Aerodrome. NICE guidance suggests Rapid Sequence Induction (RSI) is achieved within 45 minutes from 999 call where indicated and clearly the location of the base has significant implications on the ability to achieve that. Here we investigate the location of major trauma incidents in order to challenge the efficiency of the current model.

**Method**

Trauma Audit & Research Network (TARN) data was gathered on all patients admitted to Wessex Hospitals that are served by the South Central Ambulance Service (SCAS) with injury severity scores (ISS) greater than 15 over a 10 month period. Traumatic deaths were included whilst secondary transfers were not. TARN data was matched with that held by SCAS and incidents plotted geographically using specialist software. (Active Total Solutions Mapping). Isochrones were calculated to evaluate which incidents could be reached in less than 20 minutes by car.

**Results**

Matching was achievable in 367 out of the 398 patients whom met the inclusion criteria. Of these, it was possible to map 340 incidents. Only 7 incidents could be reached by car within 20 minutes of the base whilst 159 occurred within a corridor covering the M27 motorway. All incidents fell within a 15-minute flight time.

**Conclusions**

By combining incident location with isochromic distances it can be demonstrated that positioning the RRV between the 2 major conurbations of the area, up to 69% of all incidents could be reached by road within 20 minutes. This provides valuable evidence for the RRV base station to be moved to a more strategic location.

## 8. Analysis & Evaluation of the use of Whole Blood Compared to Blood Component Therapy in the Resuscitation of Traumatic Haemorrhage

### Thomas J Parker^1^, Joe L McAleer^2,3^, Kathryn L Newell^4^

#### ^1^Medical Student, Lancaster Medical School, Lancaster University, Lancaster United Kingdom; ^2^General Surgery Registrar, Northwest Deanery, Manchester United Kingdom; ^3^Clinical Anatomy Teaching Fellow, Lancaster Medical School, Lancaster University, Lancaster United Kingdom; ^4^Academic Clinical Fellow, Lancaster Medical School, Lancaster University, Lancaster United Kingdom

##### **Correspondence:** Thomas J Parker (t.j.parker@lancaster.ac.uk)

**Background**

The aim of this review is to evaluate the benefits of using Fresh Whole Blood (FWB) in the resuscitation of traumatic haemorrhage compared to the standard treatment of Component Blood Therapy (CBT). A review of the literature suggests that the change from FWB to CBT in the 1960s/70s was based upon data extrapolated from elective surgery which is rarely exposed to the same biological mechanisms seen in traumatic haemorrhage. [1] Further evidence suggests that CBT is a more anaemic, coagulopathic and thrombocytopenic product when compared to FWB, resulting in a decreased potential for coagulation and oxygenation of affected tissues. [2]

**Method & Results**

An extensive literature search found 5 studies of significance that met the evidence criteria. Three of the studies showed FWB as having a statistically significant benefit when compared to CBT, one study showed a positive correlation but the results were not statistically significant, and the final study showed no statistical difference between FWB and CBT.

**Conclusion**

This literature review concluded that the research indicated FWB could be superior to CBT in the resuscitation of traumatic haemorrhage. However, a wider research pool is required to make a definitive judgement on which treatment is the most beneficial to patients and will increase survival rates in the management of traumatic haemorrhage.

**References**

1. Goforth CW, Tranberg JW, Boyer P, Silvestri PJ. Fresh Whole Blood Transfusion: Military and Civilian Implications. Critical Care Nurse. 2016;36(3):50-7.

2. Murdock AD, Berséus O, Hervig T, Strandenes G, Lunde TH. Whole Blood: The Future of Traumatic Hemorrhagic Shock Resuscitation. Shock. 2014;41:62-9.

## 9. Time to Pre-hospital Emergency Anaesthesia (TT-PHEA): A Review of National Helicopter Emergency Medical Service (HEMS) Performance

### Jake Turner^1^, Sebastian Bourn^2^, James Raitt^3^, Erica Ley^4^, Gareth Davies^5^, Matthew O’Meara^6^

#### ^1^Anaesthetic Registrar, Nottingham University Hospitals NHS Trust, Nottingham, UK; ^2^Anaesthetic Registrar, Royal Infirmary Edinburgh, NHS Lothian, Edinburgh, UK; ^3^Emergency Medicine Registrar, Oxford University Hospital Foundation Trust, Oxford, UK; ^4^Critical Care Paramedic, Essex & Hertfordshire Air Ambulance, Colchester, UK; ^5^Emergency Medicine & PHEM Consultant, Royal London Hospital, London, UK; ^6^Anaesthetic & PHEM Consultant, University Hospital North Midlands, Stoke-on-Trent, UK

##### **Correspondence:** Jake Turner (jake.turner3@nhs.net)

**Background**

The national confidential enquiry into patient outcome and death in 2007 identified that one in eight trauma patients arrived at hospital with a partially or completely obstructed airway [1]. Subsequently, the 2017 national institute for clinical excellence major trauma guidelines recommend that patients requiring emergency anaesthesia have this performed as soon as possible, within 45 minutes of the initial call and preferably at the scene of the incident [2].

**Methods**

The pre-hospital trainee operated research network (PHOTON) retrospectively recorded TT-PHEA across all 20 eligible UK HEMS organisations, consisting of 52 enhanced care teams from1 April 2017 – 31 March 2018). The emergency call time, dispatch, arrival and paralytic drug administration times were recorded, allowing analysis of time to dispatch, travel time, time to drugs and overall TT-PHEA.

**Results**

A total of 1755 PHEA were performed by HEMS units across the UK during the period under review. The majority of PHEA were undertaken by helicopter response (67%) during daylight working hours. Median TT-PHEA was 55 minutes with 25% cases receiving PHEA within 45 minutes. The median time to dispatch (eight minutes), travel time (20 minutes) and time to administration of paralysing agent (22 minutes) were all positively skewed. Ground assets were statistically faster across all time variables compared with air assets. An increase in dispatch time from six to nine minutes correlated with a five-fold (45% to 9%) reduction in the proportion of cases for which PHEA was undertaken within 45 minutes, highlighting the importance of timely dispatch.

**Conclusions**

Marginal gains with respect to time to drugs and travel time may reduce TT-PHEA, but most crucially, efforts should be focused on reducing time to dispatch. A prospective national database of PHEA is essential for improving patient care and undertaking robust research into this field.

**Acknowledgements**

Thank you to Professor David Lockey, all PHOTON local and regional study investigators, PHOTON committee members, the FPHC RCSEd, the Association of Air Ambulances and the National Ambulance Service Medical Directors group for their support, advice and commitment to this project.

**References**

1. NCEPOD - Trauma: Who Cares? Report (2007) [Internet]. [cited 2017 Dec 6]. Available from: http://www.ncepod.org.uk/2007t.html

2. Major trauma: assessment and initial management | Guidance and guidelines | NICE [Internet]. [cited 2017 Dec 6]. Available from: https://www.nice.org.uk/guidance/ng39

## 10. Spinal injury: appreciating the problem in PICU

### Owen Morgan, Soumendu Manna

#### Paediatric Intensive Care Unit, St George’s Hospital, London, UK

##### **Correspondence:** Owen Morgan (owenmorgan1991@gmail.com)

**Background**

Epidemiology of spinal cord injury UK children is unknown. Understanding the magnitude of spinal cord injury and related outcome data can help to prognosticate as well as apportion resources appropriately. We undertook the study to assess the incidence, severity and outcome of spinal injury in children in the UK.

**Methods**

The Trauma Audit & Research Network (TARN) database was interrogated for all patients aged <16 from 2011-16. Patients with Cervical Spine Injury with or without other spinal injury (CSI) and other spinal injury (SI) were identified. Multiple data points were obtained including: Injury Severity Score (ISS), individual abbreviated injury score (AIS) and length of stay (LOS).

**Results**

15,814 patients were eligible; 381 (2.4%) patients had a CSI and 688 (4.3%) had SI. Mortality with CSI was 11.5% and SI was 2.5%, all patient mortality was 2.4%. 2695 (17%) patients required PICU admission; 151/381 (40%) of CSIs and 165/688 (24%) of SIs. CSIs admitted to PICU had a significantly higher mortality than SI (18.5% vs. 6% p=0.006). All spinal injuries are more common in children aged 11-16: CSI - 49% and SI -65%.

In children with CSI, PICU admission was associated with significantly mortality (18% vs. 7% p=0.0005) and median LOS (18 (6-51) vs. 6 (4-21) p=0.003). 92% of children with a spinal AIS of 1 had a head AIS of >2. Significant CSI was associated with significant thoracic and abdominal injuries. In children with SI mortality was significantly higher in patients admitted to PICU (6% vs 1.3% p=0.06).

**Discussion**

These results show that there is a significant burden of spinal injuries in UK children. They have high mortality and resource utilisation. Consensus on appropriate acute and long-term management based on spinal injury severity is lacking. A prospective observational study on management and outcome would inform appropriate care and resource provision for patients.

## 11. Pre-operative fasting times in orthopaedic trauma; fractured neck of femur patients

### Stephanie Kirby, Amy Davis, Emily Corbett

#### Orthopaedic Department, Southampton General Hospital, Southampton, UK

##### **Correspondence:** Stephanie Kirby (stephanie.kirby@uhs.nhs.uk)

**Background**

Currently there are no pre-operative fasting regimes for emergency patients. A decreased fasting time is associated with increased patient comfort, hydration and decreased postoperative insulin resistance^[1]^. We identified a group of patients, particularly the elderly, fasting for prolonged periods prior to their surgery.

**Method**

Standards for the audit included a maximum 8 hour fast without liquids and a 10 hour fast without food. A two-cycle audit was completed. Firstly, data collected from the emergency orthopaedic theatre list over one week for fractured neck of femur patients; identifying prolonged fasting times. Subsequent introduction of a 200ml pre-operative nutritional drink, delivering 100 calories and prescribed on the morning of theatre. The following year the standards were re-audited over a one-month period.

**Results**

The first cycle included 32 patients, of which 80% had documentation. 80% had a fasting time for solids greater than 10 hours (mean 13 hours 36 minutes) and 84% greater than 8 hours for liquids (mean 11 hours 33 minutes). The second cycle included 43 patients, 67% receiving the drink. 47% had a fasting time greater than 10 hours and 20% greater than 8 hours (mean of 6 hours 51 minutes).

Malnutrition Universal Screening Tool (MUST) scoring showed 23% of patients were at risk of malnutrition.

**Discussion**

Excessive fasting times identified in a cohort with a ‘at risk’ MUST score of 23%. Whilst this group may be at increased risk of delayed gastric emptying, stress and comorbidity it appears that the fasting times are excessive in comparison to elective patient guidelines ^[1].^ After the introduction of our nutritional drink, fasting times reduced. The new protocol requires further time to be embedded into practice and therefore prescribed for all fractured neck of femur patients. This could be expanded to other trauma cohorts of patients.

**Reference**

1. Brady M, Kinn S, Stuart P, Ness V. Preoperative fasting for adults to prevent perioperative complications. *Cochrane Database Systematic Review*. 2003;(4):CD004423.

## 12. Ventilation strategies in prehospital trauma patients: a literature review

### Hassan Khan^1^, John E McKenna^2^

#### ^1^Barts and The London School of Medicine, London, UK; ^2^Department of Anaesthesia, Royal London Hospital, London UK

##### **Correspondence:** Hassan Khan (khan.hassan50@gmail.com)

**Background**

Prehospital Emergency Anaesthesia (PHEA) is a common procedure performed in trauma patients for a number of indications [1]. From critical care literature, we know that ventilation strategies have an impact on the clinical course of major trauma patients, particularly those with severe head injuries [2] and chest injuries [3]. The prehospital environment presents particular challenges in ventilating patients. Optimal ventilation strategies for patients in this setting are yet to be determined.

**Methods**

A systematic search of the PubMed and EMBASE databases was carried out to identify studies focusing on ventilation for trauma patients in the prehospital phase of care from January 1990 to April 2019. Only original research papers with adult participants that were published in English language were included.

**Results**

A total of 18 papers were included in the qualitative synthesis and the overall quality of evidence was low. 11 papers focused on ventilation practice in traumatic brain injury (TBI), with 5 papers looking at major trauma patients in general. The prevalence of hyperventilation and hypoventilation in TBI was found to be high, indicating poor ventilation performance. 1 paper examined the use of a novel technique for titrating oxygen delivery and 1 paper researched compliance with ARDS.net protocols. 16 papers included civilian populations and 2 papers included military critical care transfer patients.

**Discussion**

There is limited evidence on ventilation strategies for trauma patients in prehospital care. The evidence that does exist generally has a narrow focus on a particular aspect of ventilation, the control of carbon dioxide. Further research in areas such as minute ventilation, positive end-expiratory pressure (PEEP), patient positioning, FiO_2_ and ventilator technology is needed to step closer to bringing targeted critical care ventilation strategies to the point of injury.

**References**

1. Burgess MR, Crewdson K, Lockey DJ, Perkins ZB. Prehospital emergency anaesthesia: An updated survey of UK practice with emphasis on the role of standardisation and checklists. Emerg Med J. 2018 Sep 1;35(9):532–7.

2. Muizelaar JP, Marmarou A, Ward JD, Kontos HA, Choi SC, Becker DP, et al. Adverse effects of prolonged hyperventilation in patients with severe head injury: A randomized clinical trial. J Neurosurg. 1991;75(5):731–9.

3. Brower RG, Matthay MA, Morris A, Schoenfeld D, Thompson BT, Wheeler A. Ventilation with lower tidal volumes as compared with traditional tidal volumes for acute lung injury and the acute respiratory distress syndrome. N Engl J Med. 2000 May 4;342(18):1301–8.

## 13. An economic analysis of open tibial fracture management at the Leeds General Infirmary (LGI)

### Sanita K Sandhu, Emily SK Coyne

#### University of Leeds, Leeds, West Yorkshire, UK

##### **Correspondence:** Sanita K Sandhu (ll13s23s@leeds.ac.uk)

**Background** Open tibial fractures (OTF) are expensive operations associated with numerous complications and a high cost per capita [1]. There is a move to review these operations and make them cost-effective in current economic austerity [1]. This study is the first to report the cost and clinical outcomes of OTF repairs at the Leeds General Infirmary (LGI) to suggest cost-saving measures.

**Methodology** A retrospective case-control study of all adult trauma patients requiring OTF repair at the LGI between April 2015 and June 2019 was conducted. Case patients were defined as those who underwent frame fixation and flap coverage on the same day. Control patients had their frame and flap on separate days. Salvage patients were excluded. Cost data for each patient’s “episode” of care was retrieved, as was data for clinical variables.

**Results** We identified 8 cases and 29 controls. Overall, same day ‘fix and flap’ was more cost-effective than delayed ‘fix and flap’ by an average of £8,108 (p<0.00497). Same day patients spent 10 fewer days in hospital post-surgery and had 6 fewer follow-up appointments. No medical or surgical related complications were reported in this group, bar superficial pin site infections which were common in both groups (87.5% vs 96.5%, respectively). No secondary procedures were performed in patients undergoing same-day management.

**Conclusion** Same day management of open tibial fractures has cost-saving potential with similar, if not better clinical outcomes. However, the relationship between infection rates, length of stay and overall cost warrants further attention. Additional research is required; there is potential for a prospective, multi-centre case-control study with a larger sample size to increase validity.

**Acknowledgements**

Leeds Teaching Hospital Trust costing team, Mr Daniel Wilks, Mr Paul Harwood, Mr Jay Wiper

**Reference**

1. Townley W, Urbanska C, Dunn R, Khan U. (2011). Costs and coding-free-flap reconstruction in lower-limb trauma. Injury [Internet]. 2011 Apr [cited 2019 Oct 19];42(4):381-4. Available at: https://www.ncbi.nlm.nih.gov/pubmed/21145546 [Accessed 19 Oct. 2019].

## 14. Elderly rib fractures in the district general hospital. More Haste, Less Death?

### Thomas Hirst^1^, Jonathan Leung^2^

#### ^1^Anaesthetic Department, East Kent Hospitals University Foundation Trust, Kent, UK; ^2^Emergency Department, East Kent Hospitals University Foundation Trust, Kent, UK

##### **Correspondence:** Thomas Hirst (thomas.hirst@nhs.net)

**Background**

Rib fractures are a commonly encountered traumatic injury and often require hospital admission and analgesia to permit adequate ventilation. Patients over 65 are recognised to be at increased risk of morbidity and mortality from rib fractures [1], despite incurring lower energy mechanisms of injury. This may be due to lack of early recognition of traumatic injuries.

**Methods**

Retrospective audit of patients with rib fractures presenting to three emergency departments over a 5-year period February 2014-February 2019. Patients were identified through TARN.

**Results**

646 patients were identified from a total of 5174 TARN entries. The majority (60.99%) of patients were aged over 65 and had a slightly lower mean injury severity score (ISS) (11.27 versus 13.25, p=0.0002). However, these patients waited significantly longer to be seen by a doctor (mean time 96.3 minutes versus 51.94 minutes, p<0.0001) and longer for CT scanning (mean time 649 minutes versus 275 minutes, p=0.0469). In patients with ISS >15, the time to see a doctor for patients over 65 was still significantly longer (mean time 76.59 minutes versus 33.64 minutes, P=0.0001). Overall mortality was 4.8%, rising to 6.85% for patients over 65. There was a trend towards lower mortality when seeing a doctor within 60 minutes (RR 0.6793, 95% CI 0.3407 to 1.3546, NNT 53.559) and performing CT scanning within 180 minutes (RR 0.7917, 95% CI 0.3935 to 1.5929, NNT 85.5). 27.1% patients over 65 were admitted to medical or geriatric wards compared with 2.38% patients under 65, a difference of 24.78% (P<0.0001).

**Conclusions**

Patients over 65 sustained the majority of rib fractures and waited significantly longer for clinical review and investigation. A significant number were initially admitted to non-surgical wards, potentially representing an initial non-traumatic working diagnosis. Strategies to promptly detect traumatic injuries from nonclassical presentations may help limit mortality in the elderly population.

**Reference**

1. Bergeron E, Lavoie A, et al. Elderly Trauma Patients with Rib Fractures Are at Greater Risk of Death and Pneumonia. J Trauma. 2003; 54(3): 478-485

## 15. A mixed methods examination of how senior paramedics determine a futile resuscitation for cardiac arrest patients in pulseless electrical activity

### Ali Coppola^1,2^, Dr Sarah Black^3^

#### ^1^Paramedic, South Western Ambulance Service NHS Foundation Trust, Exeter, UK; ^2^School of Health Professionals, University of Plymouth, Plymouth, Devon, UK; ^3^Head of research, audit and quality, South Western Ambulance Service NHS Foundation Trust, Exeter, UK

##### **Correspondence:** Ali Coppola (alison.coppola@swast.nhs.uk)

**Background** Pulseless electrical activity is a type of non-shockable cardiac arrest [1]. Paramedics treat cardiac arrests using advanced life support resuscitation [2]. When resuscitation fails, evidence on when to stop is limited [3]. This led to one UK Ambulance Service developing a local guideline to enable senior paramedics to cease resuscitation when considered futile. How senior paramedics decide futility for pulseless electrical activity is unexplored. This study aimed to investigate the clinical, patient and system factors which determined resuscitation futility and the autonomous decision-making of senior paramedics.

**Methods** An explanatory sequential mixed method study was conducted. The quantitative phase identified a consecutive sample of cessation data from 2015-2018. Data was analysed using descriptive univariate analysis. The qualitative phase identified an expert sample of six senior paramedics. Semi structured interviews were conducted and data was analysed using content framework analysis.

**Results** Quantitative phase: A sample of 50 patients had resuscitation ceased following senior paramedic involvement. Patient factors found an average age of 78 years, 74% witnessed cardiac arrest and 60% received bystander life support. Clinical factors identified 44% received defibrillation and 16% achieved an intermittent pulse. System factors found an average resuscitation duration of 54 minutes. To explore these findings in the qualitative phase, six senior paramedics were interviewed and four themes emerged: concepts that defined futility, the impact of decisions, conflicting views and tools to support decision-making. The overarching theme found senior paramedics applied clinical judgement whilst incorporating a multifactorial approach to determine resuscitation futility.

**Conclusion** Senior paramedics determined resuscitation futility by cautiously interpreting patient, system, clinical and subjective factors, including clinical judgement, to ensure decisions are holistic and appropriate to individual circumstances. It is a recommendation of this study that future cessation of resuscitation guidelines for cardiac arrest patients with pulseless electrical activity consider this multifactorial, patient centred, approach.

Acknowledgements

The authors would like to thank Professor Jonathan Benger for his valuable input and guidance as the educational supervisor for this study. J. Lynde and H. Trebilcock for quantitative data extraction. L. Tremayne and E. Freeman, qualitative data coding. Thank you to all the paramedics who participated.

**References**

1. Beun L, Yersin B, Osterwalder J, Carron P. Aetiologies of pulseless electrical activity in out- of-hospital cardiac arrests: A retrospective study and analysis of specific causes Etudiant Co-tuteur. 2014. Available from: https://serval.unil.ch/resource/serval:BIB_5590948FA30B.P001/REF. Date accessed 21 Feb 2019.

2. Perkins G, Jacobs I, Nadkarni V, Berg R, Bhanji F, Biarent D, et al. Prehospital Resuscitation [Internet]. Vol. 96, UK Resuscitation Council. 2015. p. 328–40. Available from: https://linkinghub.elsevier.com/retrieve/pii/S0300957214008119. Date accessed 23rd Nov 2018.

3. Keller S, Halperin H. Cardiac arrest: the changing incidence of ventricular fibrillation. Curr Treat Options Cardiovasc Med [Internet]. 2015 Jul;17(7):392. Available from: http://www.ncbi.nlm.nih.gov/pubmed/25981196. Date accessed​ 25 Jan 2019.

## 16. Preparation for the next major incident: are we ready? Comparing major trauma centres and other hospitals

### Jamie A Mawhinney^1^, Henry W Roscoe^2^, George AJ Stannard^3^, Sophie R Tillman^3^, Nick Freemantle^4^, T DA Cosker^2^

#### ^1^Academic Department of Vascular Surgery, Cardiovascular Division, St Thomas’ Hospital, London, UK; ^2^Nuffield Orthopaedic Centre, Oxford University Hospitals NHS Trust, Oxford, UK; ^3^Medical Sciences Division, University of Oxford, Oxford, UK; ^4^Institute of Clinical Trials and Methodology, University College, London, UK

##### **Correspondence:** Jamie A Mawhinney (jamiemawhinney12@gmail.com)

**Background**

A major incident is any emergency requiring special arrangements by the emergency services. All hospitals are legally required to keep a major incident plan (MIP) detailing the response to such events [1]. In 2006 and 2019 we assessed the confidence of key individuals in hospitals across England and found a substantial knowledge gap in responding to the MIP [2, 3]. In this study, we analyse the data from our 2019 report and compare responses from doctors at major trauma centres (MTC) to other hospitals (non-MTC).

**Method**

As before, we identified trusts in England that received over 30,000 patients through the Emergency Department in the fourth quarter of 2016/17[3]. We contacted the on-call anaesthetic, emergency, general surgery and trauma and orthopaedic registrar at each location and asked them to answer a verbal survey of three questions assessing their confidence in using their hospital’s MIP. We then compared data from doctors at MTC and non-MTC using a multinomial mixed model.

**Results**

Response rate was 62%. 50% had read at least part of the MIP, 46.8% were confident they knew where to find it and 36% knew their role in the enactment of a plan. This is broadly similar to 2006. Emergency medicine and anaesthetic registrars showed significantly higher confidence across all domains.

There was a modest difference between responses from individuals at MTC and non-MTC for question 2 (OR 2.43, CI=1.03-5.73, p=0.04) but no evidence of a difference between questions 1 (OR 1.41, CI=0.55-3.63, p=0.47) and 3 (OR 1.78, CI=0.86-3.69, p=0.12). No evidence of a systematic difference in specialty response by MTC or otherwise was identified.

**Conclusion**

Confidence in using MIPs amongst speciality registrars in England remains low. Doctors at MTC tended to be more confident but this effect was only marginally significant. We make several recommendations to improve education on major incidents.

**References**

1. HM Government. Civil Contingencies Act 2004. www.legislation.gov.uk/ukpga/2004/36/contents. Accessed 30 October 2019

2. Wong K, Turner PS, Boppana A, Nugent Z, Coltman T, Cosker TDA and Blagg SE. Preparation for the next major incident: Are we ready? Emerg. Med. J. 2006;23:709–712. doi: 10.1136/emj.2005.034025

3. Mawhinney JA, Roscoe HW, Stannard GAJ, Tillman SR and Cosker TD. Preparation for the next major incident: are we ready? A 12-year update. Emerg. Med. J. 2019 [ePub ahead of print] doi: 10.1136/emermed-2019-208436

## 17. The role of percutaneous mechanical circulatory support in patients undergoing emergency revascularisation for myocardial infarction and cardiogenic shock

### Kristina Frain^1^, Paul Rees^2^

#### ^1^Faculty of Medicine, University of St Andrews, St Andrews, UK; ^2^Academic Department of Military Medicine^2^ Barts Heart Centre, London^2^

##### **Correspondence:** Kristina Frain (Kristina.frain@ntlworld.com)

**Background**

Historically, patients requiring emergent revascularisation for acute myocardial infarction complicated by cardiogenic shock (AMI-CS) are mechanically supported by intra-aortic balloon pump (IABP) [1]. Despite advances in alternative percutaneous mechanical circulatory support devices, mortality rates in these patients remain high at approximately 50% [2]. Evidence, in support of relatively novel devices such as the Impella® is limited, and their clinical application is currently not recommended in ESC guidelines [3]. Through a comprehensive search of the available literature, this review aims to compare survival outcomes in AMI-CS patients undergoing revascularisation supported by IABP / Impella® devices and discuss the clinical implications of the findings.

**Method**

The literature was reviewed using search terms ‘Intra-aortic balloon pump’, ‘Impella’, ‘Cardiogenic shock’ and ‘Mortality’ applied to four databases: Ovid Medline, Embase, Cochrane and Web of Science. 1,823 studies were screened based on title and abstract, before full text analysis to identify studies that satisfied predefined inclusion and exclusion criteria.

**Results**

2 randomised control trials and 10 observational studies met the eligibility criteria with a total of 28,104 patients included. 2 studies investigated patient outcomes with IABP compared to Impella®. The remainder compared IABP to control. Results were mixed. Notably, only one study claimed reduced mortality with IABP vs control, and one study concluded that Impella® improved survival rates when compared to IABP. The average 30-day all-cause mortality in patients treated with IABP was 42.5% vs 37% in patients treated with Impella®.

**Conclusion**

In patients with AMI-CS undergoing revascularisation, the evidence is insufficient to associate survival benefit with treatment by IABP or Impella 2.5/CP®. When compared to IABP, the Impella 2.5/CP® was not found to provide superior survival outcomes. Whilst this analysis failed to establish a survival benefit associated with either device, the limitations of these studies have been discussed, and suggestions for future research outlined.

**References**

1. Mandawat A, Rao S V. Percutaneous mechanical circulatory support devices in cardiogenic shock. 2018;10(5):1–21. Available from: https://www-ncbi-nlm-nih-gov.access.library.miami.edu/pmc/articles/PMC5578718/pdf/nihms869918.pdf [Accessed 13 Feb 2019]

2. Rab T, O’neill W. Mechanical circulatory supprt for patients in cardiogenic shock. Trends Cardiovasc Med [Internet]. 2018;16:12. Available from: https://doi.org/10.1016/j.tcm.2018.11.014 [Accessed 11 Feb 2019]

3. Neumann F-J, Sousa-Uva M, Ahlsson A, Alfonso F, Banning AP, Benedetto U, et al. 2018 ESC/EACTS Guidelines on myocardial revascularization. Eur Heart J [Internet]. 2018;87–165. Available from: https://academic.oup.com/eurheartj/advance-article/doi/10.1093/eurheartj/ehy394/5079120 [Accessed 17 Feb 2019]

## 18. Bypassing the nearest emergency department for a more distant neurosurgical centre in traumatic brain injury patients

### Callum Prosser^1^, David Edwards^1^, Fiona Lecky^1^, Omar Bouamra^2^

#### ^1^School of Health and Related Research, The University of Sheffield, Sheffield, UK; ^2^Faculty of Biology, Medicine and Health, The University of Manchester, Manchester UK

##### **Correspondence:** Callum Prosser (Cprosser1@sheffield.ac.uk)

Background

The recent introduction of major trauma networks throughout England in 2012 has changed how patients with suspected traumatic brain injury (TBI) are managed at the scene of injury. Selecting certain head trauma patients with suspected TBI for bypass to a more distant specialist neurological centre (SNC) is the networks function but may delay resuscitation whilst expediting neurosurgical/ critical care. This comparative effectiveness research study analysed the impact of this strategy on the risk adjusted survival rates of patients confirmed to have a TBI on brain CT scan.

Method

The study employed data from the Trauma Audit and Research Network. Adult patients with a TBI on CT scan were included if they presented between June 2015 to February 2016 to SNCs or non-specialist acute hospitals (NSAH) in the North of England (South Cumbria, Lancashire and the North East Region). Patients were identified as having bypassed a nearer NSAH emergency department (ED) to a SNC using google maps enabling exclusion of patients whose nearest ED was within a SNC. Their risk adjusted survival was compared to TBI patients who received primary treatment at a NSAH with subsequent secondary transfer to a SNC or who remained at the NSAH until discharge or death. A multivariate logistic regression model predicting survival after TBI (Ps14^n^) was utilised to adjust for variation in casemix between the cohorts.

Results

84 of 339 (25%) of TBI patients bypassed a nearer NSAH to a SNC, whilst 75% received primary treatment at an NSAH (n=255). There was no significant difference in the standardised excess survival rate between the two cohorts; with +2.55% for bypass (-5.09% to +10.20%) versus -1.49% for non-bypass (-5.34% to +2.36%).

Conclusion

No significant survival benefit was identified for TBI patients who bypassed the nearest ED compared to those receiving treatment at the nearest NSAH.

## 19. Delivering balanced Red Blood Cell (RBC): Plasma transfusion in a UK air ambulance setting. Thawed fresh frozen plasma (FFP) versus freeze dried plasma

### Rachel Hawes^1,2^, Gordon Ingram^1^, Mark Cotgrave^1^, Alexander Langridge^3^, Yvonne Scott^4^, Aimi Baird^4^, Scott Rowan^4^, Jonathan Wallis^5^

#### ^1^Great North Air Ambulance Service, Stockton on Tees, UK; ^2^Department of Anaesthesia, Newcastle upon Tyne Hospitals NHS Foundation Trust, Newcastle, UK; ^3^Health Education England North East, Manchester, UK; ^4^Transfusion Laboratory, Newcastle upon Tyne Hospitals NHS Foundation Trust, Newcastle UK; ^5^Department of Haematology, Newcastle upon Tyne Hospitals NHS Foundation Trust, Newcastle UK

##### **Correspondence:** Rachel Hawes (Rachel.hawes@nuth.nhs.uk)

**Background**

Evidence for trauma haemorrhage resuscitation supports aggressive prevention of coagulopathy. This can be achieved with early balanced transfusion of RBC:thawed FFP as demonstrated in recent PROMMT [1], PROPPR [2] and PAMPer [3] trials.

Questions remain as to how to achieve this in the UK prehospital setting. FFP requires thawing, cold storage similar to RBC, costs £28/unit and has a 5 day post-thaw shelf life. Freeze dried plasma has an extended shelf life, is stored at room temperature, requires reconstitution, costs approx. £300/vial without a UK licence.

**Methods**

The Great North Air Ambulance Service has daily cool box deliveries of 2 RBC (Jan 2015) then 2RBC:2FFP (May 2016) to their 2 bases. Unused products are returned to the hospital for use or discarded at 5 days. We aimed to identify whether these clinical benefits could be reproduced with acceptable levels of wastage and cost.

Prospective data was collected between Jan 2015–Dec 2018 on patients with SBP< 90 & ongoing haemorrhage, receiving prehospital transfusion. Retrospective data was collected 12 months prior to RBC introduction.

**Results**

Cohorts matched in sex, age, MOI, injury severity. Patients received saline only (n=68), RBCs only (n=92), or RBC:FFP (n=80). 28 day mortality for saline versus FFP:RBC; odds ratio 6.78 (CI >2.22 < 20.56), P< 0.0009. No transfusion complications occurred. 12 month FFP unit usage; 66 used, 1100 returned and used in hospital, 294 not used and discarded, total cost of £28 x (66 +294) = £10,245.60. The equivalent cost of freeze dried plasma being £300 x 66 = £19,800.

**Conclusion**

Pre-thawed FFP is logistically feasible, safe, and associated with a statistically significant improvement in mortality.

Although using thawed FFP is associated with inherent wastage, it has been shown to be more cost effective than using freeze-dried alternatives and more practical to deliver in the pre-hospital setting.

**References**

1. Holcomb JB, del Junco DJ, Fox EE, et al. The prospective, observational, multicenter, major trauma transfusion (PROMMTT) study: comparative effectiveness of a time-varying treatment with competing risks. JAMA Surg. 2013;148(2):127-36.

2. Holcomb JB, Tilley BC, Baraniuk S, et al. Transfusion of plasma, platelets, and red blood cells in a 1:1:1 vs a 1:1:2 ratio and mortality in patients with severe trauma: the PROPPR randomized clinical trial. JAMA. 2015;313(5):471-82.

3. Sperry J, Guyette F, Brown J, et al. Prehospital Plasma during Air Medical Transport in Trauma Patients at Risk for Hemorrhagic Shock. N Engl J Med 2018; 379:315-326.

## 20. Geographic pattern of stab injuries presenting to a semi-rural air ambulance service: is there a link with county lines?

### M. Gavrilovski, J. Griggs, R. Lyon, C. Leahy

#### Air Ambulance Kent, Surrey & Sussex, Rochester, Kent, UK

##### **Correspondence:** M. Gavrilovski (majag@aakss.org.uk)

**Background**

The volume of knife/sharp instrument offences has increased by 44% since the year ending March 2011[1]. The particular geographical distribution of the rise in knife crime is thought to be related to county lines ‘drug-selling gangs’ [2]. The term County Lines was first coined at the Home Office in 2013 and involves child criminal exploitation; gangs using children and vulnerable people to move drugs and money [3]. ^The aim of this study was to establish the prevalence of deliberate penetrating trauma attended by semi-rural air ambulance service (Air Ambulance Kent, Surrey & Sussex) and evaluate whether there is an increase of case load and geographical link to county lines.^

^**Method**^

^A retrospective review of Air Ambulance Kent, Surrey & Sussex database (HEMSBase) was conducted of all patient who sustained deliberate assault by sharp object over a 5-year period (January 2014- December 2018), including those who died pre-hospitally.^

^**Results**^

^A total of 309 patients were identified, of which 274 were male. 46% (n=142) were age between 26 and 45, while 108 (35%) were younger than 26, of which 12 were 16 or younger. Our service saw an average annual increase of missions related to assault by sharp object of 10.52%, with the marked annual increase of 50% between 2017 and 2018. 57% (n=175) of patients were attended at night (7pm-7am). As demonstrated by heat maps, we found that the geographical distribution of patients coincided with the perceived county lines.^

^**Conclusion**^

^This study demonstrates that the increase in penetrating trauma in the region is reflected in our case load. This is the first study in the UK to analyse geographical distribution of patients assaulted by sharp object who were attended by air ambulance along the county lines. This data could be used to further inform prevention, as well as our dispatch.^

^**References**^

1. Office of National Statistics. Crime in England and Wales: year ending June 2019. 2019; [https://www.ons.gov.uk/peoplepopulationandcommunity/crimeandjustice/bulletins/crimeinenglandandwales/yearendingjune2019]. Accessed 5^th^ November 2019

2. Grimshaw R, Ford M. Young people, violence and knives – revisiting. The evidence and policy discussion. Centre for Crime and Justice studies. 2018; [https://www.crimeandjustice.org.uk/sites/crimeandjustice.org.uk/files/Knife%20crime.%20November.pdf]. Accessed 5^th^ November 2019

3. Glover Williams A, Finley F. County lines: how gang crime is affecting our young people. Archives of Disease in Childhood. 2019; 104**:**730-732

## 21. The impact of road traffic accidents on a major trauma centre in North West of England

### Andrew Hunter, Ben Rodgers, Daniel Wise, Tobias Chanin, Simon Mercer

#### Liverpool University Hospitals NHS Foundation Trust, Liverpool, UK

##### **Correspondence:** Andrew Hunter (andrew.hunter14@nhs.net)

**Background** The trauma team in a major trauma centre is resource and personnel rich [1]. A significant number of trauma team activations in England are as a result of motor vehicle accidents [2]. This service evaluation aimed to review the impact of vehicle incident related trauma team activations at a major trauma centre in the North West of England.

**Methods** Following institutional approval CAMS 6956 we reviewed all patients who activated a trauma call in the period of 1 January 2017 to 31 July 2019 recorded on our in-house trauma database. Those involved in a vehicle incident were identified and a case notes review was undertaken to record demographics and services required.

**Results** There were a total of 3912 trauma team activations in the study period of which 1226 (31.3%) where coded as ‘Vehicle Incident/Collision’ (4 were incomplete and discarded leaving 1222 for analysis). A ‘code red trauma call’ was activated in 47/1222 (3.8%) cases. The mean age was 41.2 years (SD 19.3) and 929/1222 (76.0%) were Male. During the primary survey 1081/1222 (88.4%) underwent a CT scan, 103/1222 (8.4%) required surgery within 4 hours of admission (22 trauma laparotomies) and 147/1222 (12.0%) were admitted to critical care. Head injury was documented in 569/1222 (46.6%), 115/1222 (9.4%) patients required a length of stay >3 days and 459/1222 (37.6%) were discharged on the day of admission.

**Conclusion** Vehicle incidents accounted for around one third of trauma team activations and made up a sizeable amount of work in the emergency department including CT scans as part of the primary survey. Fewer patients required urgent surgery and critical care admission than expected and almost 2/5 were discharged on the day of admission. This information is useful in planning services as often members of the trauma team have additional roles in the organisation.

**References**

1. Mercer SJ, Kingston EV, Jones CPL. The Trauma Call. *British Medical Journal* 2018; 361: 410-413

2. Moran CG, Lecky F, Bouamra O, et al. Changing the System – Major Trauma Patients and Their Outcomes in the NHS (England) 2008-17. EClinicalMedicine 2018; **2-3**: 13-21

## 22. Trauma Tertiary Survey Proforma improves tertiary survey documentation at a major trauma centre

### Apichaya Amrapala, Safiya Hafeji, Kate Hancorn, Ross Davenport, Anne Weaver

#### Department of Trauma Surgery, Royal London Hospital, London, UK

##### **Correspondence:** Apichaya Amrapala (a.amrapala@nhs.net)

**Background**

The trauma Tertiary Survey Examination (TSE) is crucial for identification of injuries otherwise missed on primary and secondary surveys and improves outcomes for trauma patients [1]. Advanced Trauma Life Support (ATLS) guidelines recommend TSE within 24-48 hours of hospital admission to include a complete head-to-toe examination with re-evaluation of previous imaging [2]. We conducted an audit of TSE of trauma patients at a Major Trauma Centre to assess whether they meet the ATLS standards and how this changed after implementing a dedicated TSE proforma.

**Methods**

TSE documentation for 30 patients retrospectively selected at random from January 2019 trauma admissions at the Royal London Hospital were evaluated for 1) time for completion of TSE, 2) completeness of head-to-toe examination and 3) review of imaging. TSE Proforma was developed and implemented in March 2019 with re-audit of 30 patients performed in June 2019. Data was analysed using chi-squared test, with significance set at p<0.05.

**Results**

29 patients had documented TSE in January, 30 patients in June. 93% of TSEs in June were completed with the proforma. Proforma use led to significant improvements in documentation of Glasgow Coma Scale (10% versus 100%, p<0.0001), imaging review (17% versus 93%, p<0.0001), central nervous system (83 % versus 100%, p=0.0226), pelvis (66% versus 100%, p = 0.0007), peripheral nervous system (66% versus 96%, p = 0.0034) and spine (83% versus 100%, p=0.0226) examinations. There was no change in TSE completion times with proforma use (76% versus 86%, p=0.35).

**Conclusion**

Use of the proforma significantly improves head-to-toe examination rates and imaging review. Not all healthcare professionals utilised the TSE proforma, suggesting further efforts are required in its implementation. Our audit recommendations will be re-audited locally in 2020 with a view to audit pan-london trauma centres.

**References**

1. Biffl, W. L., Harrington, D. T., & Cioffi, W. G. Implementation of a tertiary trauma survey decreases missed injuries. The Journal of Trauma, Injury, Infection, and Critical Care. 2003; 54(1), 38-44.

2. American College of Surgeons. ATLS Advanced Trauma Life Support Student Course Manual. 10th ed. Chicago: American College of Surgeons; 2018.

## 23. Management of penetrating chest trauma: a retrospective cohort study

### Aksha Ramaesh^1^, Elaine Teh^1^, Douglas West^1^, Gianluca Casali^1^, Eveline Internullo^1^, Rakesh Krishnadas^1^, Tim Batchelor^1^, Douglas West^1^, Thompson J^2^, Walton B^2^

#### ^1^Bristol Royal Infirmary, Department of Thoracic Surgery, Bristol, United Kingdom; ^2^Severn Major Trauma Network, Southmead Hospital, Bristol, United Kingdom

##### Correspondence: Aksha Ramaesh

**Background**

Penetrating chest trauma following assault is becoming more prevalent in the UK. In haemodynamically unstable patients, thoracotomy allows for rapid control of haemorrhage. Currently, there is limited guidance on the management of haemodynamically stable patients admitted with penetrating chest trauma. The aim of this study was to review the management of penetrating trauma in this region.

**Methods**

This is a retrospective review of patients admitted with penetrating chest trauma to Bristol from June 2014 – April 2019. Data was collected from the Trauma and Audit Research Network (TARN) database. Simple descriptive analysis was carried out.

**Results**

81cases were identified, with 61 (75%) cases admitted following assault and 14 (17%) following self-harm. 14 patients were managed under thoracic surgery. Weapons included knives, rifles, and a crossbow. There were 39 (48%) cases with ISS > 15. Major haemorrhage protocol was activated in 13 13(16%) cases.

52 (64%) patients had haemothorax, and 35 (43%) patients had pneumothorax. 5 (6%) patients had myocardial lacerations. There were 9 (11%) reported diaphragmatic injuries and 23 (28%) patients had intra-abdominal trauma.

4 cases were managed with VATS, 10 (12%) with thoracotomy and 42 (52%) with ICD. One patient required MVR, and one required embolization of a false aneurysm. The 30-day mortality was 7%. Median length of stay was 4 days (IQR 3 – 8). 49 (60%) cases were discharged home, 16 (20%) required transfer to another acute hospital, and 6 (7%) required post-operative rehabilitation.

Five patients had a post- op chest infection. One patient had post-thoracotomy pain syndrome, and one had persistent pneumothorax following ICD insertion.

**Conclusions**

Penetrating chest trauma is an increasingly prevalent presentation to ED in the UK. The majority of patients were stable on presentation and did not require transfer to a thoracic unit. Of the patients transferred to thoracics, VATS seems to be a safe approach.

## 24. Using population and demand data to inform service planning for the EMRTS Wales

### Matthew Edwards^1^, David Rawlinson^1,2^, Ami Jones^1^, David Lockey^1^ and Richard Fry^2^

#### ^1^The Emergency Medical Retrieval & Transfer Service (EMRTS) Wales, Cardiff UK; ^2^Swansea University, Swansea Wales, UK

##### **Correspondence:** David Rawlinson (david.rawlinson@wales.nhs.uk)

**Background**

The Emergency Medical Retrieval & Transfer Service (EMRTS) Cymru launched in April 2015, providing a 08:00-20:00 daily air and road response to pre-hospital emergencies and inter-hospital transfers across Wales. The Welsh Government has recently requested that EMRTS explore the options and opportunities to expand the service.

**Aims and objectives**

To determine the optimal solution that will ensure that the expansion of the EMRTS is aligned to the agreed key investment objectives in order to make a recommendation to Welsh Government.

**Methods**

A short list of service delivery options were agreed by the project team and subjected to a robust evaluation and ranking process against the key investment objectives of ‘Equity’, ‘Health Gain’, ‘Value for Money’, ‘Clinical and Skills Sustainability’ and ‘Affordability’. Data was collated from a demand versus utilisation data-matching exercise for each of the shortlisted options along with drive (road) and flight time (air) isochrones) and population coverage.

Each option’s performance was then scored and ranked against each of the 5 objectives, stakeholders were also asked to agree a weighting for each objective (reflecting its importance to their stakeholder group) in order to also prepare a weighted score per option.

**Results**

The robust process referred to (above) identified a preferred option that, in addition to the existing 12 hour 0800-2000 service provided from 3 bases, includes 24 hour bases in Caernarfon and a South Wales base, a 1400-0200 service to meet the main peak of unmet demand and extension of the Air Support Desk to support the service during the extended operating hours.

**Conclusion**

A service expansion review document has now been prepared and submitted to Welsh Government with a recommendation that the preferred option is approved and funded.

## 25. Assessing the “unmet demand” for the Emergency Medical Retrieval & Transfer Service (EMRTS) in Wales

### Matthew Edwards^1^, David Rawlinson^1,2^, Ami Jones^1^, David Lockey^1^

#### ^1^The Emergency Medical Retrieval & Transfer Service (EMRTS) Wales, Cardiff, UK; ^2^Swansea University, Swansea Wales, UK

##### **Correspondence:** David Rawlinson (david.rawlinson@wales.nhs.uk)

**Background**

The Emergency Medical Retrieval & Transfer Service (EMRTS) Cymru launched in April 2015, providing a 08:00-20:00 daily air and road response to pre-hospital emergencies and inter-hospital transfers across Wales. It is a collaborative partnership between NHS Wales, Wales Air Ambulance Charitable Trust (WAACT) and Welsh Ambulance Service NHS Trust (WAST).

**Aims and objectives**

To ascertain if there is “unmet demand” during and outside of current operational hours and to understand the timing and geographical spread of any such demand.

**Methods**

Utilising multi-source retrospective data from WAST, Trauma Audit and Research Network (TARN) and service records, a baseline demand matrix was generated detailing the average volume of calls by hour and local health board. It was corrected for the proportion of cases that would be expected to receive critical care interventions based on service records and compared with historical demand for the current service model. Data was then classified under the broad categories of Trauma, Medical and Time Critical Transfers (all Ages).

**Results**

The data reveals significant unmet demand throughout the 24 hour period, with the majority between 15:00 and 21:00, but also significant numbers outside of current operational hours. The focus of the unmet demand is proven to be in the South East of Wales.

**Conclusions**

There is a significant unmet need, and the service should develop a case for expansion that utilises the above information and identifies the optimal solution in line with agreed key investment objectives.

## 26. Trauma in undergraduate medical education - are we doing enough?

### Aditi Nijhawan^1^, Shadman Aziz^2^, Jonathan Martin^1^, Joyce Kam^1^, Lewis Forrester^1^, Sam Thenabadu^1^

#### ^1^GKT School of Medical Education, King’s College London, UK; ^2^Barts Health NHS Trust, London UK

##### **Correspondence:** Aditi Nijhawan (adi.nijhawan@gmail.com)

**Background**

Junior doctors are often the first clinicians to assess multiply-injured trauma patients [1], however teaching in trauma is traditionally done at postgraduate level [2]. A significant number of soon-to-graduate medical students in the past have believed their trauma training to be sub-par [3], with similar beliefs reflected in junior doctors [1].

The King’s College London (KCL) Pre-hospital Care Programme (PCP) is a student-led initiative open to all KCL medical students, giving them the opportunity to experience pre-hospital care with the aim of furthering their exposure to acutely unwell medical and trauma patients.

**Methods**

Students applying to the programme were asked to complete a ‘pre-PCP’ survey on their previous exposure to emergency medicine and trauma, and confidence in patient assessments using Likert scales. Students will be re-surveyed following their participation in the PCP, as part of a wider study assessing the influence of pre-hospital care experience on students’ clinical practice.

**Results**

36 student responses were analysed; all had completed at-least one year of medical school. 58% of respondents said they had moderate to extensive exposure to acutely unwell patients, with only 31% saying the same for patients with traumatic injuries. Similarly, while 30% respondents were confident or extremely confident in performing ABCDE assessments on medical patients, only 14% felt the same about performing a trauma primary survey.

**Discussion**

There is a clear disparity between students’ experiences of traumatic and medical presentations, likely reflecting the gap in trauma exposure and training at undergraduate level that has regularly been reported. Effective assessment of an unwell patient is a key part of the GMC ‘outcomes for graduates’; the KCL PCP aims to develop students’ exposure to and understanding of trauma patient assessment, and hence facilitate a more confident transition to foundation years.

KCREC Ethical Clearance Reference: MRS-18/19-13553

**References**

1. Price A, Hughes G. Training in advanced trauma life support. BMJ [Internet]. 1998 [accessed 10 November 2019];316(7135):878-880. Available from: https://www.bmj.com/content/316/7135/878.short

2. Carley S, Driscoll P. Trauma education. Resuscitation [Internet]. 2001 [accessed 10 November 2019];48(1):47-56. Available from: https://www.sciencedirect.com/science/article/pii/S0300957200003178?via%3Dihub

3. Mastoridis S, Shanmugarajah K, Kneebone R. Undergraduate education in trauma medicine: The students’ verdict on current teaching. Medical Teacher. 2011;33(7):585-587.

## 27. A retrospective audit of pre-hospital hypotension and hypoxia in severe traumatic brain injury

### Dr Sarah Kennie^1^, Dr Tim Nicholson Roberts^1,2^, Dr Simon Hughes^2^

#### ^1^Neurosciences Intensive Care Unit, University Hospital Southampton NHS Foundation Trust, Tremona Rd, Southampton, UK; ^2^Hampshire and Isle of Wight Air Ambulance, University Hospital Southampton NHS Foundation Trust, Tremona Rd, Southampton, UK

##### **Correspondence:** Sarah Kennie (sarah.kennie@uhs.nhs.uk)

**Background**

Traumatic brain injury (TBI) is the commonest cause of death in those <40 years old in England and Wales [1]. Hypotension (systolic blood pressure (sBP) <90mmHg) and/or hypoxia (SaO2 <90%) in the patient with a TBI has been associated with increased risk of secondary brain injury and mortality [2, 3]. The injured brain is especially vulnerable in the pre-hospital phase, patients with severe TBI (GCS ≤8) therefore benefit from Helicopter Emergency Services (HEMS) care and pre-hospital emergency anaesthesia (PHEA).

**Methods**

We carried out a retrospective audit on all adults (≥18 years) with severe TBI who were attended to by Hampshire & Isle of Wight Air Ambulance (HIOWAA) between 1/11/2018 and 26/9/2019. Data was collected from the ARC-EMS patient record database. Cases with documented hypotension and/or hypoxia were examined in detail, we focused on oxygenation and haemodynamics pre and post PHEA where applicable.

**Results**

33 patients had severe TBI, those in cardiac arrest without evidence of return of spontaneous circulation (ROSC) were excluded. 12 (36%) cases of hypotension and/or hypoxia were identified. Of these, 4 (33%) occurred during HEMS primary survey only, the other 8 (64%) occurred subsequent to this whilst under HEMS care. 21/33 (64%) received PHEA. Post PHEA hypotension and hypoxia rates were 15% and 5% respectively. The mean change in sBP following RSI was -15mmHg. sBP reduction post PHEA was most marked in polytrauma patients and the elderly.

**Conclusion**

Patients with severe TBI were at risk of PHEA induced hypotension and airway compromise associated hypoxia. Suggested changes to practice included: increased use of vasoactive drugs (particularly during induction of anaesthesia) as well as more scrupulous attention to the documentation of observations and clinical decision making. Recent changes to ARC-EMS, now with automated data pull through, will add scope to a re-audit.

**References**

1. Lawrence T, Helmy A, Bouamra O, Woodford M, Lecky F, Hutchinson P. Traumatic brain injury in England and Wales: prospective audit of epidemiology, complications and standardised mortality. BMJ Open. 2016;6(11):e012197.

2. Spaite D, Bobrow B, Keim S, Barnhart B, Chikani V, Gaither J et al. Association of Statewide Implementation of the Prehospital Traumatic Brain Injury Treatment Guidelines With Patient Survival Following Traumatic Brain Injury. JAMA Surgery. 2019;154(7):e191152.

3. Spaite D, Hu C, Bobrow B, Chikani V, Sherrill D, Barnhart B et al. Mortality and Prehospital Blood Pressure in Patients With Major Traumatic Brain Injury. JAMA Surgery. 2017;152(4):360.

## 28. Isolated thoracic trauma in patients admitted to an UK Urban Major Trauma Centre: epidemiology and outcomes

### Isabella Wilkes^1^, Christopher Aylwin^1,2^, Elaine Cole^2^

#### ^1^St Mary’s Hospital, London UK; ^2^London Major Trauma System, London UK

##### **Correspondence:** Isabella Wilkes (isabellamarywilkes@gmail.com)

**Background**

Major trauma has traditionally affected young males, however an aging population has increased the proportion of older trauma patients. Mechanisms are changing where low level falls are common in older people and violent trauma is increasingly affecting younger cohorts. Currently age-related thoracic guidance is not routinely available, and the burden and outcomes of isolated thoracic major trauma have not been described in the UK.

**Methods**

A retrospective review of prospectively collected data at an urban Major Trauma Centre (MTC) was performed. Data between February 2014 and December 2018 was sourced from the Trauma Audit Research Network database. Patients aged ≥16 years with isolated thoracic trauma, irrespective of chest abbreviated injury score (AIS) and injury severity (ISS) were included. Injury characteristics, treatment and outcomes (mortality and Glasgow Outcome Score) were compared between younger (<60yrs) and older populations (≥60yrs),

**Results**

Of the 419 patients with isolated thoracic trauma, 54% were younger and 46% older. Chest AIS and ISS did not differ between ages (AIS 3[3-4], ISS 9 [9-16] both groups). Older adults had a 2-fold increase in ≥3 rib fractures (77% vs. 38%, <0.0001), and were three-times more likely to suffer flail chest injuries (36% vs. 13%, <0.0001). Haemopnuemothoraces were twice as frequent in the young (37% vs. 17%, <0.0001). There was a four-fold increase in surgery for younger patients (29% vs. 7%, p<0.001), whereas epidural rates doubled for older adults (30% vs. 17%, p 0.001). Mortality did not significantly differ (5% vs 9%, p=0.12) but more than a quarter of older patients were moderately disabled at discharge (28% vs 4%, p<0.0001).

**Conclusion**

Despite comparable injury severity, isolated thoracic trauma at a UK MTC differed between age cohorts. Whilst the majority survived irrespective of age, treatment pathways differed. Further national investigation into isolated thoracic trauma is warranted to enhance specific age-related clinical guidance.

## 29. The impact of gunshot wounds at a major trauma centre in the north west of England

### Rebecca Russell, Simon J Mercer

#### Liverpool University Hospitals NHS Foundation Trust, Liverpool, UK

##### **Correspondence:** Simon J Mercer (simonjmercer@hotmail.com)

**Background** Penetrating trauma is less common than blunt trauma in major trauma centres in England [1] and as a consequence the trauma team has less exposure to such cases. In the year ending March 2017 there were 6,375 firearm offences recorded in England & Wales [2]. The objective of this service evaluation was to review the impact of gunshot wounds (GSW) on our major trauma centre over a three-year period and to review the resources that were required and to assess the impact on our training programs.

**Methods** Following institutional approval (CAMS 7113) we undertook a retrospective service evaluation of patients who triggered a trauma call as a result of a GSW during the period 1 August 2016 to 31 July 2019. Cases were identified using our in-house trauma database and a case notes review was undertaken. Demographic data and any services utilised were recorded.

**Results** There were 4483 trauma calls over the study period of which only 84 (1.8%) were attributed to GSW (1 record was incomplete and discarded). The mean age was 30.1 years (SD 11.0), 80/83 (96.4%) were Male and 66/83 (79.5%) presented outside normal working hours (0800-1800). A Code Red Trauma Call was activated for 28/83 (33.7%), CT Scan was performed for 57/83 (68.7%), 12/83 (14.5%) required surgery within 4 hours of admission (7 trauma laparotomies), 10 patients were admitted to critical care, 5 died and 27 patients were admitted for > 7 days.

**Conclusions** There were relatively few gunshot wounds but when they presented a third required a ‘code red trauma’ activation and arrived predominately out of normal working hours. These patients are resource intensive and there are training implications as they present infrequently. High-fidelity simulation could be considered as part of a training package to supplement the lack of exposure to these cases.

**References**

1. Moran CG, Lecky F, Bouamra O, et al. Changing the System – Major Trauma Patients and Their Outcomes in the NHS (England) 2008-17. EClinicalMedicine 2018; **2-3**: 13-21

2. Firearm Crime Statistics: England & Wales. Available at: https://researchbriefings.parliament.uk/ResearchBriefing/Summary/CBP-7654 (accessed 8 November 2019)

## 30. Implementation of a rapid response vehicle for emergency medical retrievals and transfers during the twilight period across South Wales

### Julia Parnell^1^, David Rawlinson^2^, Ami Jones^2^

#### ^1^Welsh School of Anaesthesia, Health Education and Improvement Wales, Cardiff, Wales, United Kingdom; ^2^Emergency Medical Retrieval and Transfer Service Cymru, Cardiff, Wales, United Kingdom

##### **Correspondence:** Julia Parnell (julia.parnell1@nhs.net)

**Background**

The Emergency Medical Retrieval and Transfer Service (EMRTS Cymru), launched in April 2015, provides Pre-hospital critical care for all age groups by conducting time critical care and life or limb threatening transfers to specialist centres. It allows rapid intervention and transfer through Rapid Response Vehicles or Air Ambulance coverage, between 08:00-20:00, daily across Wales.

Demand for further provision was recognised outside of commissioned hours, especially during the pressurised winter months. Therefore, to support frontline ambulance and hospital services in South Wales, EMRTS Cymru introduced an additional critical care car-based service - the ‘Twilight Critical Care Car’. With funding from the Welsh Government, this service aimed to provide treatment to patients whom the current twelve-hour operations would not be available.

**Method**

Working alongside the existing service, an additional Critical care team in an EMRTS Rapid Response Vehicle was available between the hours of 14:00-02:00. Available every Friday, Saturday and Sunday for a three month time period between January and March 2019, the service, based at Cardiff Heliport, provided identical critical care to the routine helicopter service, covering the South East and South West of Wales.

**Results**

A total of 36 shifts were provided over the three month period, with 278 tasked calls, 89 of which were out of current EMRTS operational hours. Of the 60 patients requiring admission, 68% bypassed district general hospitals to provide faster access to specialist definitive care. Concordance with predicted workload was favourable and will be further tested as the service expands.

**Conclusion**

Successful implementation demonstrated a demand for additional critical care services, suggesting room for expansion of the current hours conducted by EMRTS to assist the emergency services in South Wales. Case studies identified timely stabilisation with direct access to larger specialist facilities, reducing the impact of secondary transfers on district general hospitals.

## 31. The effect of a Trust-wide awareness campaign on quality and outcomes after admission with rib fractures

### Ji Hye Moon, Shu Ning Yew, Elaine Teh, Timothy Batchelor, Gianluca Casali, Eveline Internullo, Rakesh Krishnadas and Douglas West

#### Department of Thoracic Surgery, University Hospitals Bristol NHS Foundation Trusts, Bristol UK

##### **Correspondence:** Ji Hye Moon (jihye.moon@doctors.org.uk)

**Background**

Rib fractures are common, and associated with significant morbidity and mortality. Early assessment and analgesia are important, but were variably provided in our hospital. We assessed the effect of a trust wide awareness campaign on assessment, management and outcomes.

**Methods**

All patients admitted to the thoracic surgery service with rib fractures between April 2017 - March 2018 (audit cycle 1) and June - September 2019 (cycle 2) were included. Demographics, clinical management, documentation of rib fracture score calculation and length of stay were collected. Data were compared with the first audit cycle before the Trust awareness campaign. Fisher’s exact, Student’s 2 tailed t tests and Mann-Whitney U tests were used as appropriate.

**Results**

56 patients were available for analysis. Higher rib fracture scores were associated with increasing age and longer length of stay. In the post intervention group, the rib fracture score calculation rate has improved from 0% to 50% and the pain management compliance rate from 11% to 71%. In addition, mean resting pain scales ranged from 3.72 (day 1) to 3.52 (day 5), suggesting generally good quality pain control.

**Conclusion**

A Trust wide initiative significantly improved the early assessment of rib fracture patients and compliance with management protocols. Increases in the use of regional anaesthesia or surgery were not significant, but pain control appeared adequate. Implementation did not therefore require significantly increased surgical or anaesthetic resources.

## 32. Correction of traumatic brain injury induced coagulopathy using fluids and blood products

### Samuel Smith^1^, Paul Vulliamy^2^, Ross Davenport^2^

#### ^1^University of Liverpool School of Medicine, Liverpool, UK; ^2^Centre for Trauma Sciences, Queen Mary University of London, London, UK

##### **Correspondence:** Samuel Smith (hlssmi10@liv.ac.uk)

**Background**

Traumatic Brain Injury (TBI) frequently results in an acute coagulopathy [1] which is associated with a substantial increase in mortality [2,3]. The aim of this study was to examine the effect of fluid and blood product transfusions on the correction of TBI-induced coagulopathy, and to investigate the clinical relevance of persistent uncorrected coagulopathy.

**Method**

This retrospective study used data from the Activation of Coagulation and Inflammation in Trauma Study (ACIT). Patients with an isolated TBI (iTBI: head AIS ≥3 and other AIS components <3) and an admission coagulopathy (ExTEM CA5 ≤40mm on ROTEM analysis or INR >1.2) were included. Patients who remained coagulopathic at 24 hours were compared to patients whose coagulopathy resolved.

**Results**

Of 55 coagulopathic iTBI patients, 34 had their coagulopathy corrected and 21 remained coagulopathic. The groups were comparable in terms of overall and intracranial injury severity, but persistently coagulopathic patients had a greater admission base deficit (0.2 vs 2.3, p=0.022) and a higher incidence of coagulopathy by admission INR (4% vs 35%, p=0.008). Patients with a persistent coagulopathy needed significantly more neurosurgical decompression (18% vs 43%, p=0.041) and received more red blood cells (0 vs 1 units, p=0.006). However, volumes of crystalloid, fresh-frozen plasma, platelets and cryoprecipitate administered were similar, and the majority of patients in both groups did not receive any blood products by 24 hours. Although mortality was comparable, multiple organ dysfunction was more frequent among persistently coagulopathic patients (62% vs 86%, p=0.06).

**Discussion**

Persistent coagulopathy occurs in a substantial proportion of coagulopathic iTBI patients and is associated with a higher level of shock and higher rates of neurosurgical intervention and organ dysfunction. Although we did not find evidence that aggressive blood product resuscitation is associated with correction of coagulopathy, use of products was infrequent in this study and further investigation is required.

**References**

1. Greuters S, van den Berg A, Franschman G, Viersen VA, Beishuizen A, Peerdeman SM, et al. Acute and delayed mild coagulopathy are related to outcome in patients with isolated traumatic brain injury. Critical care (London, England). 2011;15(1):R2.

2. Kunio NR, Differding JA, Watson KM, Stucke RS, Schreiber MA. Thrombelastography-identified coagulopathy is associated with increased morbidity and mortality after traumatic brain injury. American journal of surgery. 2012;203(5):584-8.

3. Epstein DS, Mitra B, O'Reilly G, Rosenfeld JV, Cameron PA. Acute traumatic coagulopathy in the setting of isolated traumatic brain injury: a systematic review and meta-analysis. Injury. 2014;45(5):819-24.

## 33. Review of the first 20 prehospital blood product transfusions by Essex and Herts Air Ambulance (EHAAT)

### T Durham^1^, I Norris^1^, L Philipson^2^

#### ^1^Faculty of Medicine, University College London, London, UK; ^2^Essex and Hertfordshire Air Ambulance, Earls Colne, UK

##### **Correspondence:** I Norris (Isabel.norris.14@ucl.ac.uk)

**Background**

Life threatening haemorrhage is the leading cause of preventable death amongst trauma patients [1]. Prehospital blood products have been used by an increasing number of civilian helicopter emergency medical services [1]. EHAAT introduced plasma and packed red cells into service in March 2019.

**Methods**

We investigated the first 20 cases of prehospital blood product administration by EHAAT using retrospective analysis of the HemsBASE 2.0 database. We conducted a survey of EHAAT clinical staff to review experiences of blood product administration and reviewed the EHAAT standard operating procedure for blood product administration.

**Results**

Analysis demonstrated a similar patient demographic receiving blood product to other UK HEMS services [2] [3]. 75% of patients were male, and 11/20 patients were aged between 20-40 years. The predominant mechanism of injury was road traffic collision, reflecting the urban-rural area of EHAAT coverage.

EHAAT were 100% compliant with pre-transfusion checklists and blood traceability practice. There was no wastage of packed red cells and one unit of lyoplas (plasma) was reconstituted but not given.

Our analysis demonstrated that patients in traumatic cardiac arrest were treated off standard operating procedure and given larger volumes of packed red cells without plasma. This reflects a need for large volume resuscitation. 6/13 non-cardiac arrest patients were also given packed red cells prior to plasma potentially reflecting a clinical decision to give larger volumes. However difficulties were identified with the time taken to reconstitute plasma and this warrants further audit.

**Acknowledgements**

Essex and Hertfordshire Air Ambulance (EHAAT)

**Conclusion**

Blood product transfusion by EHAAT has been shown to be logistically sound and safe. Further audit of blood product administration by EHAAT is required.

**References**

1. Shand S, Curtis K, Dinh M, Burns B. What is the impact of prehospital blood product administration for patients with catastrophic haemorrhage: an integrative review. Injury. 2018 Dec 12

2. Raitt JE, Norris-Cervetto E, Hawksley O. A report of two years of pre-hospital blood transfusions by Thames Valley Air Ambulance. Trauma. 2018 Jul; 20(3):221-4

3. Rehn M, Weaver A, Eshelby S, Lockey D. London's air ambulance: 3-year experience with pre-hospital transfusion. Resuscitation. 2015 Nov 1; 96:156

## 34. Assessing the research impact of London’s Air Ambulance in the field of pre-hospital medicine

### Alexandra Valetopoulou^1^, Maria Ahmad^1^, Michael D Christian^2^

#### ^1^Barts and the London School of Medicine and Dentistry, London, UK; ^2^London Air Ambulance, Royal London Hospital, London, UK

##### **Correspondence:** Michael D Christian (michael.christian1@nhs.net)

**Background**

London’s Air Ambulance (LAA) provides advanced trauma care across London, bringing specialist resources and expert trauma teams to patients. Since its inception 30 years ago, LAA has treated 40,000 critically injured patients [1] and significantly impacted pre-hospital patient care. To our knowledge, this is the first analysis to quantify the magnitude of the research impact of LAA. We aim to provide an insight into the research impact that LAA has had in pre-hospital medicine since its establishment.

**Methods**

We searched Scopus, Web of Science, Google Scholar and Pubmed using pre-determined key words. Subsequently the references list of each article was reviewed to identify additional articles not included within the search. Articles were deemed LAA focused and were thus included if they were regarding pre-hospital medicine and if: (a) data were derived from LAA or, (b) focused on LAA patients or, (c) authored by members of LAA. The research impact of each eligible article was classified as either: high *(article directly influenced the change/creation of clinical guidelines)*; medium *(article was referenced in clinical guidelines or has >20 Google Scholar citations, or >10 Pubmed citations)*; or low impact *(article has ≤20 Google Scholar citations or ≤10 Pubmed citations).*

**Results**

The literature search yielded 1,120 articles in total. 200 articles met our inclusion criteria and their full text was analysed to determine the level of impact. 19 articles were classified as high impact, 76 as medium impact and 105 as low impact. Of the 76 articles classified as medium impact, 20 were referenced in guidelines but not thought to have significantly influenced a change in the guideline.

**Conclusion**

This review quantifies the significant research impact of LAA in pre-hospital medicine over the past 30 years and considers its future potential.

**Acknowledgments**

Thank you to Sister Liz Foster for her valuable contribution.

**Reference**

1. London’s Air Ambulance Charity [Internet]. 2019 [accessed 10 November 2019]. Available from: https://www.londonsairambulance.org.uk

## 35. Pre-Alert Calls in Major Trauma patients conveyed to Trauma Units

### Thomas Hirst^1^, Helen Sheriff^2^

#### ^1^Anaesthetic Department, East Kent Hospitals University Foundation Trust, Canterbury, UK; ^2^Critical Care Paramedic, South East Coast Ambulance Service, Crawley, UK

##### **Correspondence:** Thomas Hirst (thomas.hirst@nhs.net)

**Background**

At present there are no clear guidelines governing pre-alert calls for trauma patients outside of the major trauma decision tree, which makes recommendations for patients suitable for direct conveyance to the Major Trauma Centre (MTC) and recommends pre-alert in select groups. We sought to investigate the use of pre-alert calls in patients conveyed to 2 trauma units.

**Methods**

Retrospective audit of trauma patients conveyed to 2 trauma units in Kent between February 2014 and February 2019. Patients were identified through TARN.

**Results**

835 major trauma patients (ISS >15) were identified, of whom 278 (33.29%) were pre-alerted to the trauma unit. 258 patients had a trauma team activation with 222 (79.86%) pre-alerted prior to arrival. 96 (11.5%) patients had GCS <14, of whom 53 (55.2%) were pre-alerted These patients had mortality of 18.75%, with a relative risk of death of 2.95 (95% CI 1.79 to 4.86, P <0.0001), NNH 8.01 compared with GCS≥14. 110 patients (17.37%) required secondary transfer to the major trauma centre of whom 57 (51.82%) were pre-alerted.

**Conclusions**

Prehospital alerts accounted for the majority of hospital trauma team activations in our cohort of ISS >15 patients. However, the majority of major trauma patients were not pre-alerted. Where pre-alert calls are traditionally performed for patients assessed to need immediate intervention, we would encourage calling in all patients where significant injuries are suspected to allow for immediate assessment. The activation of a trauma team allows more rapid clinical review, expedited imaging, discussion with the MTC and even an earlier recognition and discussion of futility in frail multiply injured patients. We also identified that the major trauma decision tool was often not followed for patients with GCS <14 (step one) where pre-alert is always recommended, and these patients had significantly poorer outcomes.

## 36. Pre-hospital frailty scoring by critical care teams in older patients with traumatic injury: is it feasible?

### Kurtis Poole, James Raitt, Dhushy S Kumar, Syed Masud

#### Thames Valley Air Ambulance, Stokenchurch, Buckinghamshire, UK

##### **Correspondence:** Kurtis Poole (kurtis.poole@tvairambulance.org.uk)

**Background**

Older patients are increasingly sustaining traumatic injury. Those aged 60 and over account for more than half of patients recorded in the Trauma Audit and Research Network database.[1] The assessment of frailty may guide clinical decision making for these patients.

Pre-hospital critical care teams offer enhanced care and senior decision making at the point of injury. These teams are exposed to information that is not available to hospital teams that may assist frailty assessment.

The Clinical Frailty Scale (CFS) is a judgement-based tool to assess frailty ranging from one, ‘very fit’, to nine, ‘terminally ill’ [2].

This service evaluation aimed to review the feasibility of obtaining a pre-hospital CFS for patients attended by a critical care team.

**Methods**

Thames Valley Air Ambulance provides physician and paramedic critical care teams to a population of over two million people. Data was collected from the 9th April 2019 to the 9th October 2019. The attending team recorded a CFS or an inability to assess on the patient record. Cases were included where the patient was aged 60 or older and had experienced an acute traumatic injury.

**Results**

There were 156 eligible cases retrieved. Six cases were missing all data and were excluded.

Of the remaining 150 cases, 143 recorded a CFS (95.3%). This included nine of ten patients who received a pre-hospital anaesthetic (90%). The proportion of very frail patients (CFS > 6) was directly proportional to age.

**Discussion**

The pre-hospital assessment of frailty is highly feasible in older trauma patients. There are numerous potential benefits such as guiding interventions, hospital destination, and in-hospital specialist review. Frailty scores are collected in medical cases, and likely offer similar utility. Reproduction of this work in similar settings, and further evaluation of the validity and impact of pre-hospital frailty scoring, is warranted.

1. Banerjee J, Baxter M, Coats T, Edwards A, Griffiths R, Kumar D, et al. Major Trauma in Older People. 2017 https://www.tarn.ac.uk/content/downloads/3793/Major%20Trauma%20in%20Older%20People%202017.pdf Accessed 1 Nov 2018.
2. Rockwood K, Song X, MacKnight C, Bergman H, Hogan DB, McDowell I, et al. A global clinical measure of fitness and frailty in elderly people. CMAJ. 2005;173(5):489–95.

## 37. End tidal versus blood gas for measurement of carbon dioxide in the prehospital setting: a service evaluation

### Jonathan Hall^1^, Charlotte Thompson^2^, Tim Godfrey^1,2,3^

#### ^1^University of Bristol, School of Medical Sciences, Bristol, UK; ^2^Emergency Department, North Bristol NHS Trust, Bristol, UK; ^3^Great Western Air Ambulance Charity), Bristol, UK

##### **Correspondence:** Charlotte Thompson (Charlottethomps87@gmail.com)

**Background**

Maintenance of low normocapnia (4-4.5kPa) in the context of traumatic brain injury (TBI) is crucial for neuroprotection. [1] The end tidal carbon dioxide (ETCO_2_) level is routinely taken as a proxy of arterial CO_2_ concentration (PaCO_2_) in the prehospital environment as blood gases are not easily measurable, despite PaCO_2_ being the gold standard. [2] It has been suggested that in the prehospital population ETCO_2_ cannot be used reliably as a surrogate for PaCO_2_. [3] The aim of this project was to determine the reliability of using ETCO_2_ as a surrogate for arterial PaCO_2_ in our local prehospital TBI population.

**Methods**

A retrospective service evaluation was performed, in which twenty pre-hospital cases were identified who had potentially suffered a TBI and were given a prehospital emergency anaesthetic. The differences between the last prehospital and first in-hospital ETCO_2_ measurements and the first in-hospital PaCO_2_ measurement were calculated. Patients who did not have PaCO_2_ measured in the Emergency Department were excluded from the analysis.

**Results**

Sixteen patients were included in the analysis. The median prehospital and in-hospital ETCO_2_ levels were almost identical: 3.8kPa and 3.85kPa respectively. The median PaCO_2_ was 5.3kPa on arrival in hospital. ETCO_2_ levels were consistent across all patients with very little variation away from 4kPa, whereas the PaCO_2_ results were noticeably more varied, ranging from 4.2kPa to 7.8kPa. In four cases there was a >2 kPa difference between ETCO_2_ and PaCO_2_.

**Conclusions**

ETCO_2_ is an unreliable surrogate measurement for arterial PaCO_2_ in the prehospital setting. Given that the median PaCO_2_ was 5.3kPa for a corresponding median ETCO_2_ of 3.8kPa, true arterial PaCO_2_ may be significantly over the recommended upper limit for patients with suspected TBI. The ability to measure prehospital arterial blood gases would allow for more accurate PaCO_2_ levels to be targeted and maintained. Further work would be a prospective observational study in this area.

**References**

1. Dinsmore J. Traumatic brain injury: an evidence-based review of management. Continuing Education in Anaesthesia Critical Care & Pain. 2013; 13(6):189-95.

2. Waldmann C, Soni N, Rhodes A. Oxford Desk Reference Critical Care. Oxford: Oxford University Press; 2008.

3. Belpomme V, Ricard-Hibon A, Devoir C, Dileseigres S, Devaud ML, Chollet C, et al. Correlation of arterial PaCO_2_ and ETCO_2_ in prehospital controlled ventilation. Am J Emerg Med. 2005; 23(7):852–9.

## 38. Adequacy of vascular access in pre-hospital major haemorrhage

### Anthony Hudson, Maja Gavrilovski

#### Air Ambulance Kent Surrey Sussex (AAKSS), Redhill Aerodrome, Redhill, Surrey, UK

##### **Correspondence:** Anthony Hudson (anthonyh@aakss.org.uk)

**Background**

Vascular access in the context of major trauma is a key intervention that facilitates a number of important interventions, not least volume resuscitation in the context of major haemorrhage. Current NICE guidance recommends the use of peripheral vascular access for circulatory access and if this fails to consider intraosseous access in the pre-hospital setting^1^. There is no guidance should this be unachievable or inadequate.

**Method**

A retrospective review of all “Code Red” (major haemorrhage) cases attended to by AAKSS between 2013 to 2018 was conducted to identify what vascular access was identified on primary survey and subsequently inserted by the HEMS team.

**Results**

A total of 85 patients (28%) had no vascular access on arrival of the HEMS team. Of these intravenous access as achieved with only a single cannula in 13 patients (4%) and 5 (1.5%) were transferred to hospital with only intraosseous access. A total of 63 patients (21%) arrived at hospital with less than 2 points of intravenous access. Detailed review revealed several patients were managed with only small gauge intravenous access, some required attempts at central access and there were 2 cases of hypovolaemic arrest with inadequate access cited in both cases.

**Discussion**

In line with NICE guidance^1^ all patients were managed with either peripheral vascular access or intraosseous access. However there a small but significant group of patients who are managed in the pre-hospital phase with inadequate access and this can lead to adverse outcomes including significant hypovolaemia. It is advisable for pre-hospital teams to be trained in and carry equipment to facilitate central venous access.

1. Trauma: assessment and initial management. NICE guideline (NG39). February 2016. https://www.nice.org.uk/guidance/ng39/chapter/recommendations#management-of-haemorrhage-in-prehospital-and-hospital-settings (Date accessed 16^th^ July 2018)

## 39. Adjusted 30-day mortality is not significantly affected by direct transport to a Major Trauma Centre after traumatic brain injury (TBI): A single-centre retrospective observational cohort study

### John Eraifej^1,2^, Jonathan E. Attwood^2^, Tim Lawrence^2,3^

#### ^1^Nuffield Department of Surgical Sciences, University of Oxford, Oxford, UK; ^2^Department of Neurological Surgery, John Radcliffe Hospital, Oxford, UK^; 3^Nuffield Department of Clinical Neurosciences, University of Oxford, Oxford, UK

##### **Correspondence:** John Eraifej (John.Eraifej@ouh.nhs.uk)

**Background**

Effective management must combine measures to minimise secondary brain injury with urgent neurosurgical intervention when indicated [1]. However, the mortality of TBI remains high, and the optimal pre-hospital transport strategy for TBI within the UK Major Trauma Network remains unclear [2]. We compare the effect of direct versus indirect transport to a neurosurgical centre on the mortality of patients following TBI.

**Method**

A retrospective observational cohort study of all patients admitted to a major trauma centre with neurosurgery available (MTC) within 24 hours of head injury (Abbreviated Injury Score ≥3) between 01/01/2014 – 31/12/2017. Cases missing key data fields (injury date/time, hospital admission date/time) were excluded (454 cases, 27.4%).

**Results**

843 patients were transported directly, 359 were transported indirectly (n=1202). Median time from incident to first hospital was 101.0 minutes (IQR 77.0-135) for direct transport and 97.0 minutes (IQR 66.0-178.0) for indirect transport (p=0.865, Mann-Witney U). Median time from incident to MTC admission was 101.0 minutes (IQR 77.0-134) for direct transport and 545.0 minutes (IQR 361.0-1547.0) for indirect transport (p<0.001, Mann-Witney U). After adjustment for age, sex, ISS and pre-hospital GCS, pupil reactivity, SpO2, heart rate and systolic blood pressure, odds ratio of 30-day mortality after direct transport was 0.658 compared to indirect transport (p=0.263, 95% CI 0.316–1.370, binary logistic regression).

For patients requiring neurosurgery (including intracranial pressure monitoring), there was no statistical difference in time from MTC admission to neurosurgery (n=290, p=0.360). After adjustment for age, sex, ISS and pre-hospital GCS, pupil reactivity, SpO2, heart rate and systolic blood pressure, odds ratio of 30-day mortality after direct transport was 0.373 compared to indirect transport (p=0.122, 95% CI 0.107–1.300, binary logistic regression).

**Conclusions**

After adjustment for baseline pre-hospital characteristics, there is no adverse effect of indirect transfer to an MTC on mortality. This represents appropriate local selection of patients for direct transfer.

**References**

1. McCullough AL, Haycock JC, Forward DP, Moran CG. Early management of the severely injured major trauma patient. Br J Anaesth. 2014;113:234–41.

2. Lecky F, Russell W, Fuller G, McClelland G, Pennington E, Goodacre S, et al. The Head Injury Transportation Straight to Neurosurgery (HITS-NS) randomised trial: a feasibility study. Health Technol Assess (Rockv). 2016;20:1–198.

## 40. Exploring differences between ISS and NISS scores for 30-day mortality in adult and elderly trauma patients in a Norwegian national trauma cohort

### Mathias Cuevas-Østrem^1^, Olav Røise^2^, Torben Wisborg^3^, Elisabeth Jeppesen^4^

#### ^1^Norwegian Air Ambulance Foundation, Oslo, Norway; ^2^Norwegian Trauma Registry, Division of Orthopaedic Surgery, Oslo University Hospital, Oslo, Norway; ^3^Norwegian National Advisory Unit on Trauma, Division of Emergencies and Critical Care, Oslo University Hospital, Oslo, Norway; ^4^Faculty of Health Sciences, University of Stavanger, Oslo, Norway

##### **Correspondence:** Mathias Cuevas-Østrem (mathias.cuevas-ostrem@norskluftambulanse.no)

**Background**

Injury Severity Score (ISS) and New Injury Severity Score (NISS) with a threshold over 15 is commonly used to define severe injury and to define the study population in trauma registry-based studies for both adult and elderly trauma patients (1). For elderly patients (≥65 years) this might be unreasonably high and might lead to exclusion of significantly injured elderly with increased risk of mortality. The aim of this study was to assess whether there were significant differences in 30-days mortality between adults and elderly trauma patients for different frequently used ISS and NISS thresholds

**Method**

The Norwegian Trauma Registry was interrogated to identify all adult (≥16 years) trauma patients included in the registry from January 2015 through December 2018. Data were dichotomized to age groups “adult” and “elderly” (16-64 and ≥65 respectively) with 30-days mortality as primary endpoint. Mortality rates were assessed for ISS and NISS thresholds of >9, >12 and >15. We applied descriptive statistics and Chi-squared test for comparisons.

**Results**

23768 patients with available information about age, 30-days mortality and ISS and NISS scores were included in the analysis, of which 16224 patients were 16-64 years old and 4706 patients were ≥65 years. 238 adult and 500 elderly patients died, giving overall mortality rates of 1.5% and 10.6% respectively. For ISS and NISS >9 there was a significantly higher 30-days mortality in elderly trauma patients (17.3% and 15.2% respectively) than adult patients (4.7 and 3.8% respectively) (p<0,001), as for all other ISS and NISS thresholds tested.

**Conclusions**

This study demonstrates that elderly trauma patients has a significantly higher mortality risk than adult trauma patients at all ISS or NISS-thresholds analysed. This group has a significant mortality even at ISS and NISS above 9.

**References**

Palmer CS, Gabbe BJ, Cameron PA. Defining major trauma using the 2008 Abbreviated Injury Scale. Injury. 2016;47(1):109-15.

## 41. Assessment of the usability of the Draeger VE300 prehospital ventilator by UK critical care paramedics

### Peter O. Beaumont^1^, Aidan Baron^1^, Samuel Dixon^2^, Lawrence Hayes^2^

#### ^1^Department of Paramedic Science, Kingston and St George’s University of London, London UK; ^2^South East Coast Ambulance Service NHS Foundation Trust, Crawley, Sussex, UK

##### **Correspondence:** Peter O. Beaumont (p.beaumont@sgul.kingston.ac.uk)

**Background**

During CPR, ILCOR guidelines on ventilation are rarely achieved due to a tendency to either over or under ventilate both in terms of frequency and tidal volume. Most mechanical ventilators are unable to solve this problem as they struggle to cope with high airway pressures generated during CPR. The Draeger VE300 is a novel prehospital ventilator with a ‘CPR mode’ which is pre-set to deliver ILCOR recommended ventilations as well as modulate flow during compressions in order to effectively deliver tidal volumes without excessive airway pressure. The device has the capacity to deliver additional modes of invasive and non-invasive ventilation, something not seen in other prehospital devices. The VE300 has a unique design in that it incorporates the oxygen cylinder within its housing, negating the need for a long, high pressure oxygen hose.

**Methods**

This manufacturer sponsored study aimed to assess the usability of the device by UK critical care paramedics in a single ambulance service. The primary means by which data was collected was via an electronic Ventilator Use Record Form on a bespoke App (Teamscope) completed after each use.

**Results**

Over 98 days in 2018/19 the ventilator was deployed 55 times (29 during cardiac arrest, 32 post-ROSC only). There were 2 uses in non-invasive mode. The overall impression by users was that 81.8% had a positive experience, 9.1% were neutral and 9.1% negative. 29.1% of users decided to return to BVM due to the presence of alarms related to ‘leakage’ or ‘check circuit’. 1 device suffered a screen malfunction and another a cracked screen. The integrated cylinder mechanism and fixation hooks may need revising. Despite a detailed training program users often felt underconfident troubleshooting problems, but appreciated additional clinical information on the VE300.

**Conclusion**

A majority of users felt the VE300 was worth further consideration in cardiac arrest management.

## 42. An evaluation of the interventions used in post-concussion syndrome: A systematic review

### Nisha Chauhan, Rebecca A Murray

#### Blizzard institute, Bart’s and the London school of medicine and dentistry. Queen Mary’s University of London, Bethnal Green, London, UK

##### **Correspondence:** Nisha Chauhan (Chauhan_nisha90@hotmail.com)

**Background** Post-concussion syndrome (PCS) affects 15-25% of mild traumatic brain injury (mTBI) patients resulting in a variety of incapacitating symptoms from dizziness and headache to difficulty sleeping and depression.^(1)^ This review focuses on the three interventions most used in clinical practice: rest, information giving and cognitive behavioural therapy (CBT). Rest is increasingly being questioned as a treatment in many areas of medicine but is still largely promoted in concussion management.^(2)^ Information giving is an integral part of an emergency department consultation but has not necessarily proven to help PCS symptoms. Lastly, CBT has a firm founding in psychiatry and, due to the dysfunctional thought processes and maladaptive beliefs known to underpin chronic PCS, it is likely to be an effective treatment.^(3)^ This review will look at the evidence available for all three of these interventions in the context of PCS.

**Method** Systematically reviewing studies from; MEDLINE, Embase, PsycINFO and PubMed for original, non-observation studies published from 1998-2019 including adults with mTBI in relation to the intervention of rest, CBT or information giving. Excluding sports concussion studies.

**Results** Of the 20,902 studies identified, 14 were included in this review. Three rest papers, four CBT papers and seven for information giving. There were major methodological differences with many different clinical outcome measures used. Two of the three rest studies showed no effect of rest on PCS symptoms. The CBT studies that focused on acute PCS all found the intervention effective. The information giving studies yielded inconclusive results.

**Conclusion** Of the three interventions assessed in this review only CBT has proven effective in the treatment of acute PCS. Rest does not have sufficient evidence to support its promotion. Information giving will remain an integral part of common practice, but this review has found inadequate evidence to support its use in reducing PCS symptoms.

**References**

1. Varner, C. McLeod, S. Nahiddi, N. Lougheed, R. Dear, T. and Borgundvaag, B. (2017). Cognitive Rest and Graduated Return to Usual Activities Versus Usual Care for Mild Traumatic Brain Injury: A Randomized Controlled Trial of Emergency Department Discharge Instructions. Academic Emergency Medicine, 24(1), pp.75-82.

2. Allen, C., Glasziou, P. and Mar, C. (1999). Bed rest: a potentially harmful treatment needing more careful evaluation. The Lancet, 354(9186), pp.1229-1233.

3. Brunger, H., Ogden, J., Malia, K., Eldred, C., Terblanche, R. and Mistlin, A. (2013). Adjusting to persistent post-concussive symptoms following mild traumatic brain injury and subsequent psycho-educational intervention: A qualitative analysis in military personnel. Brain Injury, 28(1), pp.71-80.

## 43. Missed opportunities for primary HEMS transfer to MTC – Isle of Wight experience

### Dr. Alex H. Oldman^1^, Dr. Foteini Dandolou^1^ and Dr. Simon Hughes^2^

#### ^1^Emergency Department, Queen Alexandra Hospital Portsmouth, UK; ^2^Hampshire & Isle of Wight Air Ambulance, University Hospital Southampton, UK

##### **Correspondence:** Alex H. Oldman (alex.oldman@gmail.com)

**Background**

The Isle of Wight (IOW) is covered by the Hampshire and Isle of Wight Air Ambulance (HIOWAA) critical care prehospital service from 07:00-02:00. The Wessex Major Trauma Network ‘Trauma Unit Bypass Tool’ (TUBT) is a set of criteria instructing ambulance paramedics regarding which patients require primary HEMS transfer to the nearest Major Trauma Centre (MTC) in Southampton, bypassing the island’s Trauma Unit (TU). Some patients are first taken by IOW road ambulance to the IOW TU and have subsequent secondary HEMS transfer to the MTC. We investigated secondary transfers for any missed primary transfer opportunities.

**Methods**

Prehospital data relating to all secondary transfers to the MTC from IOW between January 2016 and December 2017 was gathered from Trauma and Audit Research Network (TARN) and IOW Ambulance Service databases. All prehospital data was checked against the TUBT for missed primary MTC transfer opportunities.

**Results**

48 secondary transfers were identified. 23 (48%) of these did not meet the bypass tool criteria and 13 (27%) patients met the bypass tool criteria but were missed primary transfers. Recorded secondary transfer injuries included: reduced Glasgow Coma Score (n=7), systolic blood pressure <90mmHg (n=5), depressed or open skull fracture (n=2), suspected pelvic fracture (n=2), and crushing, degloving or mangled limb injury (n=1). 6 (13%) patients self-presented to the TU and 6 patient files had incomplete data. 47 (98%) met the bypass criteria within HEMS operational hours and there was no obvious geographical location bias.

**Conclusion**

27% of patients from the IOW had a missed primary transfer opportunity, having met defined criteria set out in the bypass tool. The creation of opportunities to promote wider dissemination of the tool amongst ambulance teams is needed to increase numbers of safer patient transfers off the island.

**Acknowledgements**

Thanks to UHS TARN team and the IOW ambulance service.

## 44. IDART: Impairing drug and alcohol as risk factors for traumatic injuries - preliminary results from a national study on injury prevention

### C.C. Braathen^1^, B.M. Joergenrud^2^, E. Skadberg^2,^ S.T. Bogstrand^2^, H. Gjerde^2^, L.A. Rosseland^3^, T. Kristiansen^4^

#### ^1^Department of Acute Medicine, Division Elverum-Hamar, Innlandet Hospital Trust, Oslo, Norway; ^2^Department of Forensic Sciences, Division of Laboratory Medicine, Section of Drug Abuse Research, Oslo University Hospital, Oslo Norway; ^3^Department of Research & Development, Division of Emergencies and Critical Care, Oslo University Hospital, Oslo, Norway; ^4^Department of Anaesthesiology, Division of Emergencies and Critical Care, Rikshospitalet, Oslo University Hospital, Oslo, Norway

##### **Correspondence:** C.C. Braathen (camilla.brathen@gmail.com)

**Background:** Traumatic injuries constitute one of our major public health challenges.

The most effective means to reduce the impact trauma has on individuals and society is primary injury prevention. Alcohol and drug use are major risk factors for traumatic events. Primary prevention relies on detailed knowledge of risk factors. The aim of this study is to facilitate targeted injury prevention through improved data collection and analysis on impairing substances as risk factors for traumatic injuries.

**Methods:** IDART is a national prospective study including analyses of the toxicological profile of all patients ≥ 16 year of age admitted via trauma team activation to any Norwegian trauma hospital (*n*38) during a 12 month study period. Residual blood from routinely drawn blood samples at trauma admission is analyzed for alcohol, illegal and psychoactive drugs. Toxicological data are linked to clinical data from the National Trauma Registry.

**Results:** Data collection started March 1st, 2019, and during the first six months 3006 patients were included from 34 trauma hospitals. According to preliminary data, 33% of the included patients tested positive for psychoactive substances; alcohol: 20%, illegal drugs: 9%, and prescribed psychoactive drugs: 13%. Mean alcohol blood concentration was 1,7 g/kg and cannabis was the most commonly used illegal drug. Data on the prevalence of different psychoactive substances disaggregated by mechanism of injury from the 6 month study period will be presented.

**Conclusions:** More than 30% of the included patients tested positive for one or more psychoactive substance according to preliminary data. The IDART study provides a detailed descriptive analysis on the prevalence of alcohol, illicit and medicinal drug use among all patients admitted to a Norwegian hospital with suspected severe injury. Further analyses will aim to identify high risk groups according to age, gender, geographical location, circumstances of the injury and type of psychoactive substance.

## 45. Microvesicles have increased thrombin generation potential early after trauma

### Ingrid N Rognes^1^, Marit Hellum^2^, William Ottestad^3^, Kristi G Bache^1^, Torsten Eken^3^, Carola Henriksson^4^

#### ^1^Norwegian Air Ambulance Foundation, Oslo, Norway; ^2^Institute of Clinical Medicine, University of Oslo, Oslo, Norway; ^3^Department of Anaesthesiology, Clinic of Emergencies and Critical Care, Oslo University Hospital, Oslo, Norway; ^4^Department of Medical Biochemistry, Oslo University Hospital, Oslo, Norway

##### **Correspondence:** Ingrid N Rognes (i.n.rognes@gmail.com)

**Background**

Microvesicles (MVs) are small membrane-surrounded particles that are released from various cell types in response to cell activation and apoptosis. Several studies have shown an increased number of MVs after trauma using flow cytometry [1,2]. Circulating MVs after trauma are thought to have a procoagulant potential. We hypothesised that the thrombin generating capacity of isolated MVs after injury was increased and correlated with degree of anatomical injury (New Injury Severity Score, NISS) and physiological derangement (admission base excess, BE).

**Method**

MV-associated thrombin generation was assessed in admission blood samples in a prospective cohort of 33 trauma patient, and in 20 healthy controls. We used a modified thrombin generation assay where MVs are isolated and analysed in pooled normal plasma [3]. This method measures the activity of only MVs, without contributing effect from the patients’ plasma. Wilcoxon rank sum test was used for group comparison, and Spearman correlation was used to evaluate the correlation between NISS and BE and MV associated thrombin generation.

**Results**

Citrated plasma from 33 patients and 20 controls were used for analysis. The patient population were severely injured (75% NISS ≥25, and 25% admission BE ≤ -6), with predominantly blunt injury (94%). 8 patients received ≥2 units of packed red blood cells in the ER. Compared to controls, trauma patients’ thrombin generation curves had shorter lag time (LT) (median 13.6 vs. 18.5 minutes, p<0.0001) and higher peak (median 60.7 vs. 40.9 nM, p<0.001). Admission BE correlated with both LT (Spearman rho 0.47, p<0.01) and peak (rho ‑0.35, p<0.05), whereas NISS only correlated with LT (rho ‑0.48, p<0.01).

**Conclusion**

MVs from trauma patients have a hypercoagulable potential immediately after injury. MV-associated thrombin generation parameters LT and peak had a consistent statistical correlation with physiological derangement, whereas anatomical injury severity only correlated with LT.

**References**

1. Park MS, Xue A, Spears GM, Halling TM, Ferrara MJ, Kuntz MM, et al. Thrombin generation and procoagulant microparticle profiles after acute trauma. Journal of Trauma and Acute Care Surgery. 2015 Nov; 79(5):726–31.

2. Matijevic N, Wang Y-WW, Holcomb JB, Kozar R, Cardenas JC, Wade CE. Microvesicle phenotypes are associated with transfusion requirements and mortality in subjects with severe injuries. J Extracell Vesicles. 2015; 4(1):29338.

3. Hellum M, Franco-Lie I, Øvstebø R, Hauge T, Henriksson CE. The effect of corn trypsin inhibitor, anti-tissue factor pathway inhibitor antibodies and phospholipids on microvesicle-associated thrombin generation in patients with pancreatic cancer and healthy controls. Garcia de Frutos P, editor. PLoS ONE. Public Library of Science; 2017 Sep 14; 12(9)e0184579–15.

## 46. Festival of colours and its impact on Health Care Delivery System – A 3 Year Experience at Level 1 Trauma Center in India

### Narendra Choudhary, Abhinav Kumar, Kapil Dev, Amit Gupta

#### Division of Trauma Surgery and Critical Care, Jai Prakash Narayan Apex Trauma Centre, All India Institute of Medical Sciences, New Delhi, India

##### **Correspondence:** Narendra Choudhary (narendra3483@gmail.com)

**Background:**

Holi is celebrated as the festival of colors and brotherhood in India. Traditionally people meet friends and relatives with exchange of sweets and colors. This involves more outdoor activities and travel as a part of Holi celebration with predisposition to injuries and trauma. Some Cultural and Socioeconomic groups indulge in alcohol and substance abuse on the occasion of Holi festival. Violence and defiance are inherent to substance abuse and ultimately leads to injuries and subsequent Hospital visit.

**Methods:**

A Retrospective observational study was conducted at JPN Apex Trauma Center, AIIMS, New Delhi. The Trauma Registry (Prospectively maintained data base) for duration of last 03 year, Extending from January 2017 to April 2019 was analysed.

The analysis include Total Emergency Department (ED) attendance, Triage category according to the severity of injury, and total ED Deaths on the day of Holi Celebration and compared with one day prior and one day after the Holi celebration.

**Results:**

A Total No of 494 patient arrived in ED in the duration of 01 day (24 Hours) on Holi Festival in year 2019. The Triage of patients are 275, 192 and 27 as Green, Yellow and Red area Respectively. There was no ED Death on the day.

In year 2018, the ED attendance for the same festival was 432 patients, out of them 303 patients were triaged into Green area, 101 patient in Yellow and 27 patients in Red. There was a ED Death on the same day and triaged as Black.

In Year 2017, Total 402 patient arrived in ED on the Holi Festival and Triaged as 240, 125 and 36 patient in Green, Yellow and Red Area Respectively. 01 ED Death was Triaged as Black.

**Conclusion:**

Holi Festival is associated with mass casualty situation with a magnitude to overwhelm the existing healthcare facility. Disaster associated with Holi is unique as Holi is celebrated on a prefixed date according to Hindu Calendar and stake holders are well aware in advance about the incoming mass casualty situation unlike other disasters.

The situation can be improved with stringent law enforcement against substance abuse. General awareness programmes addressing the ill effect of substance abuse should be implemented among public.

## 47. Cardiac reperfusion injury following resuscitative thoracotomy: a review

### Zoe Leadbetter^1^, Ben Singer^2^

#### ^1^The Institute of Pre-hospital Care, Whitechapel, London, UK; ^2^Barts Health NHS, London’s Air Ambulance, Queen Mary University, London, UK

##### **Correspondence:** Zoe Leadbetter (v6y18@students.keele.ac.uk)

**Background**

Resuscitative thoracotomy (RT) is indicated in a subset of traumatic cardiac arrests and can result in neurologically intact survivors [1]. It has been hypothesised that patients who achieve return of spontaneous circulation may die hours to days later due to cardiogenic shock and multiple organ dysfunction syndrome precipitated by cardiac reperfusion injury.

Complex multifactorial mechanisms underpin cardiac reperfusion injury including the oxygen, calcium and pH paradox, inflammation and mitochondrial dysfunction [2].

The aim was to identify the number of deaths following RT, for blunt and penetrating trauma, that could potentially be attributed to cardiac reperfusion injury.

**Methods**

A literature search of Medline, Embase, Scopus, Web of Science and PubMed databases was conducted to identify eligible RT case series. This was done in accordance with the preferred reporting items for systemic reviews and meta-analyses (PRISMA) guidelines. To be eligible studies must detail a case series of >5 patients who underwent a thoracotomy that was resuscitative in nature and provided sufficient details of the outcome of patients.

**Results**

Eight case series were included within the systematic review with a total of 649 patients who underwent RT. 47.7% (45/95) of late patient deaths following RT had modes of death that could potentially be attributed to cardiac reperfusion injury. This equates to 6.9% (45/649) of the total patients. Mortality in all RT patients was 94% (610/649) and early mortality (<6 hours since injury) was 79% (515/649).

**Discussion**

This review suggests a proportion of patient may die due to the consequences of cardiac reperfusion injury. There is evidence to suggest that there is a window of opportunity to mitigate the injury caused by reperfusion [3]. Interventions such as withholding calcium chloride, partial rather than total aortic occlusion, and early assessment for possible extra-corporeal mechanical support may be beneficial however further research is required.

**References**

1. Lockey DJ, Brohi K. Pre-hospital thoracotomy and the evolution of pre-hospital critical care for victims of trauma. *Injury* 2017;48(9):1863-64

2. Sanada S, Komuro I, Kitakaze M. Pathophysiology of myocardial reperfusion injury: preconditioning, postconditioning, and translational aspects of protective measures. *American Journal of Physiology-Heart and Circulatory Physiology.* 2011;301(5):1723-41.

3. Abela CB, Homer-Vanniasinkham S. Clinical implications of ischaemia-reperfusion injury. *Pathophysiology.* 2003;9(4):229-40.

## 48. Post-traumatic pulmonary pseudocyst: a fatal case

### Alberto Tredese^1^, Giulia Roveri^1^, Mattia Gomarasca^2^, Alessandro Calzolari^2^, Sara Cucchetti^2^, Erika Borotto^2^, Danilo Radrizzani^2^

#### ^1^Department of Medical- Surgical Physiopathology and Transplants, University of Milan, Milan, Italy; ^2^Department of Intensive Care Unit, Legnano Hospital, Italy

##### **Correspondence:** Erika Borotto (erikaborotto@gmail.com)

**Background**

Post-traumatic pulmonary pseudocyst (TPP) is uncommon complication after blunt trauma due to important compressive forces transmitted to the lung parenchima: alveoli and interstitium are lacerated and cavities fulfilled with air, fluid or both are created [1].

Thoracotomy is reserved to cases of massive haemorrhage or persistent air leakage with low incidence of mortality.

**Case Report**

We report the case of a 37 years old male crashed by a S.U.V passing over his chest.

He had no past illness apart from mild obesity.

On the scene hemodynamic was unstable and tracheal intubation was performed for worsening in respiratory mechanic.

CT showed bilateral pulmonary contusions with pneumo, hemothorax and thoracic vertebral fractures requiring urgent surgical fixation.

Total Injury Severity Score was 38 and Trauma Injury Severity Score 9,9%.

ICU stay was complicated by sepsis due to pulmonary infection, severe acute respiratory distress syndrome requiring prone position and acute kidney injury due to hypermyoglobinemia. A follow-up CT revealed an air-fluid filled cavity 8 cm diameter consistent with TPP.

Patient improved until a massive hemothorax, requiring emergency thoracotomy with atypical lobar resection. After this complication was resolved, it was possible to complete respiratory weaning despite persistence of air leakage and of TPP at CT scan. On day 57 another massive hemothorax occurred together with hemoptysis inhibiting ventilation and exiting in cardiac arrest. Massive transfusion protocol was activated, broncoscopy and bronchial blocker insertion were attempted without benefit. VA-ECMO was impossible at that time.

**Conclusion**

TPP usually recover spontaneously with conservative therapy but fatal cases happen. Aggressive surgical treatment like lobar or pulmonary resection should be considered in case of haemorrhage or persistent air leakage because of high risk of dramatic evolution. Recent evidence of VA-ECMO without systemic anticoagulation could be considered as a bridge to operating room in non ventilable, hemodynamically instable patients [2].

**References**

1. Fagkrezos et al. Post-traumatic pulmonary pseudocysit with hemopneumothorax following blunt chest trauma: a case report. J Med Case Rep. 2012; 6:356

2. Wen et al. Non heparinized ecmo serves a rescue method in a multitrauma patient combining pulmonary contusion and nonoperative internal bleeding: a case report and review of literature. World J Emerg Surg 2015; 10:15

Written, informed consent for publication was obtained from the patient.

## 49. An analysis of the exposure of anaesthetic trainees to knife injuries in a Major Trauma Centre

### Jennifer English, Victoria Morgan, Simon J Mercer

#### Liverpool University Hospitals NHS Foundation Trust, Liverpool, UK

##### **Correspondence:** Jennifer English (jen2311@doctors.org.uk )

**Background** The incidence of knife crime in England and Wales has risen since 2013, with 47,000 assaults using a knife or sharp instruments being reported in the year ending 2019 [1]. This audit will assess how rising knife crime is shaping trauma training for anaesthetic trainees and will review exposure to this type of injury.

**Methods** Following approval from our institution (CAMS 7085) we identified all patients who had triggered a trauma call due to stabbing from our in-house database 1 August 2016 and 31 July 2019. We then undertook a case note review to determine data on age, gender, time of admission, operating theatre attendance and procedures undertaken.

**Results** There were 3528 trauma team activations during the study period of which 590 (16.7%) were due to stabbings. Mean age was 31.0 years (SD 11.5) and 542/590 (91.2%) were male and 391/590 (66.3%) were admitted outside of the normal working day (0800-1800). There were 69/590 (11.7%) patients who required surgical intervention within 4 hours of admission (including 31 trauma laparotomies, 3 thoracotomies and 2 combined Laparotomy and Thoracotomy). We determined that an anaesthetic registrar will cover 40 on calls during a 6-month rotation and so per shift there is a 3% chance that they will manage a patient with stab wounds requiring urgent surgical intervention.

**Discussion** Despite the increasing incidence of knife crime, it remains a small part of our trauma workload. The exposure of anaesthetic trainees to knife wounds requiring surgery through on call duties alone is limited. Supplementary methods of training such as high-fidelity simulation should be considered to provide trainees with more experience. As two-thirds of these incidents occurred outside normal working hours, undertaking twilight shifts during a dedicated trauma block may maximise exposure.

**Reference**

1 Allen G et al. Knife Crime in England and Wales. House of Commons. Briefing paper SN4304, 2019.

## 50. Imaging in Silver Trauma at the crossroads-A single center review

### Muneeba Nasir, Jonathan Leung, Sophie Parrok, Alice Wilkinson, Salman Naeem

#### Accident and Emergency Department, East Kent Hospitals University NHS foundation Trust, Ashford, Kent UK

##### **Correspondence:** Muneeba Nasir (muneeba.nasir1@nhs.net)

**Background**

Elderly Trauma accounts for more than 20% of the UK major trauma. There is a lack of evidence regarding the sensitivity of early CT in the silver trauma considering the added challenges faced in the geriatric patients.

**Methods**

This retrospective review analyzed the yield of CT (regional and/or panbody) vs X-ray as the first imaging of choice in 536 elderly (>65 years) patients registered in the TARN (The Trauma Audit and Research Network) database for a single trauma unit over a year.

**Results**

CT was requested as the first imaging modality in 57% of the patients (n-304) with 77% (n-233) regional and 23% (n-71) pan body scans. The yield of CT for positive findings was 84% for the regional and 100% for the panbody scans respectively. Regional X-ray was requested as the first imaging modality for 43% (n-232) of the geriatric trauma patients. Around 42% of these patients went on to have regional CT of the same region as initial X-ray with 74% of such scans yielding positive findings whilst the pan body CT following initial X-ray were positive in all 09 patients (100%).

**Conclusion**

Our retrospective analysis of imaging in elderly trauma for a single trauma unit concludes that we need to lower our threshold for requesting CT as the first imaging modality in the geriatric trauma patients with complex assessment challenges, thereby adopting less stringent guidelines as ED physicians and the radiologists alike.

## 51. Emergency Medical Education and Training (EMET) in the Australian outback

### Sass Hayes^1,2^, Bethany Luxford^1,2^, Mathew Davies^2^

#### ^1^Retrieval Services Queensland, Queensland Health, Australia; ^2^Australasian College for Emergency Medicine, Australia

##### **Correspondence:** Sass Hayes (sass.hayes@health.qld.gov.au)

**Background**

Emergency Medicine Education and Training (EMET) provides education, training and supervision to health professionals working in emergency departments and emergency care services – particularly in rural, regional and remote Australia – who are not specifically trained in emergency medicine (EM). These remote communities often have challenging healthcare needs amidst disparities in healthcare provision caused by significant workforce shortages and large geographical distances [1].

**Method**

The EMET Program was established in 2012, with Commonwealth Funding, to provide EM training to regional, rural and remote clinicians that is tailored to their local requirements [2]. The training is provided in the clinician’s own environment, addressing local emergency health issues, by a group of emergency medicine specialists who are Fellows of the Australasian College for Emergency Physicians (FACEMs) [3].

**Results**

Over the past 7 years FACEMs have had in excess of 127,000 attendees at training sessions across more than 500 regional, rural and remote sites, via the 49 supported EMET hubs. EMET also provides expert supervision and support for doctors working in emergency departments (EDs) to complete ACEM’s EM Certificate (EMC) or EM Diploma (EMD), which to date has produced over 1000 EMC and 50 EMD graduates. EMET has had wide ranging effects including significant improvements in patient quality of care measures, improved skills of clinical staff and more appropriate referrals from regional, rural and remote facilities to retrieval services. This has led to greatly enhanced service delivery at the supported training sites. The model has enabled the building of relationships and facilitated networking opportunities between the hub FACEMs and their colleagues at the training sites.

**Conclusion**

The ACEM EMET Program continues to provide regional, rural and remote healthcare clinicians with confidence, skills and knowledge in EM. The hub and spoke model and outreach training has enabled the building of strong relationships between all involved.

**References**

1. Pandit T, Ray R, Sabesan S. Review article: Managing medical emergencies in rural Australia: A systematic review of the training needs. Emerg Med Australas. 2019 Feb;31(1):20-28

2. Australasian College for Emergency Medicine (2017), Emergency Medicine Education and Training Guidelines, Melbourne

3. Australasian College for Emergency Medicine, Quality Standards for Emergency Departments and other Hospital-Based Emergency Care Services, Australian College for Emergency Medicine, Melbourne, 2015

## 52. Emergency Telehealth in Queensland, Australia

### Sass Hayes^1^, Clifford Pollard^2^, Daniel Best^3^

#### ^1^Retrieval Services Queensland, Queensland Health, Queensland, Australia; ^2^Jamieson Trauma Institute, Queensland Health, Queensland, Australia; ^3^Telehealth Support Unit, Queensland Health, Queensland, Australia

##### **Correspondence:** Sass Hayes (sass.hayes@health.qld.gov.au)

**Background**

Telehealth models of care have been available in Queensland Health for over 30 years, providing a vital service in overcoming many of the challenges involved in delivering rural and remote health care across a vast area [1]. The model has expanded into emergency medicine over the past decade to cover over 109 regional and remote hospitals and primary health clinics enabling clinicians to provide crucial clinical advice to their rural counterparts.

**Method**

Emergency Telehealth Models of Care include

*Existing*

- Critical Care and Retrieval Support – via Retrieval Services Queensland
- Low Acuity Emergency Support – via Telehealth Emergency Management Support Unit (TEMSU)
- Speciality Emergency eConsultation – various services including burns and dermatology
- Intrahospital Emergency Coordination – between emergency departments and theatres

*Emerging*

- TeleICU – supporting regional ICUs
- TeleStroke – videoconferencing and remote radiology imaging
- Remote surgery and procedural advice – providing support to regional operating theatres

We are now moving towards more advanced telehealth models of care that require precision advice, greater collaboration, and health care outside the traditional hospital setting, bringing the need for new features and technologies for telehealth [2]. This will include the ability to annotate, measure, manipulate and point while in a videoconference to better assist the remote clinician through a range of different procedures, or the use of portable/wearable videoconferencing technologies to provide a greater “point of view” image either in pre-hospital, acute or post-acute scenarios.

**Results**

The technology discussed in this paper has enabled the provision of a high quality critical and acute care statewide telehealth service [3]. Some of the benefits include

- Improved patient care and access to specialist support
- A more complete clinical picture to inform patient triage
- Better informed resource allocation to ensure efficient utilisation of resources
- More reliable clinical management support, and educational and training opportunities for clinicians located in rural and remote settings.

**Conclusion**

The above telehealth technologies are now an established and integral part of the day to day delivery of healthcare throughout the state of Queensland.

**References**

1. Hayiroglu Ml. Telemedicine: Current Concepts and Future Perceptions. Anatol J Cardiol. 2019 Oct;22(Suppl 2):21-222

2. Dorsey ER, Topol EJ. State of Telehelath. N Engl J Med. 2016 Jul 14;375(2):154-6

3. Emergency Medicine Telemedicine. Ann Emerg Med. 2016 May;67(5):687-9

## 53. Time to start first chest compression and return of spontaneous circulation rate in out of hospital cardiac arrest calls in Saudi Arabia

### Meshary BinHotan^1,2^, Joanne Turnbull^2^, Graham Petley^2^, Nawfal Aljerian^3^, Meshal Alanazi^4^, Mohammed Altuwaijri^4^

#### ^1^Emergency Medical Services, King Saud bin Abdulaziz University for Health Sciences, Riyadh, Saudi Arabia; ^2^Health Sciences, University of Southampton, Southampton, United Kingdom; ^3^Medical referrals centre, ministry of health, Riyadh, Saudi Arabia; ^4^Saudi Red Crescent Authority, Riyadh, Saudi Arabia

##### **Correspondence:** Meshary BinHotan (M.S.Bin-Hotan@soton.ac.uk)

**Background**

Out of hospital cardiac arrest (OHCA) is still a major health problem affecting many people worldwide with 8.6% and 10.6% survival rate in England and USA, respectively. Early initiation of cardiopulmonary resuscitation (CPR) can double or quadruple the survival rate [1]. American Heart Association (AHA) recommended callers to start chest compression in less than 180 seconds from call receipt [2] Providing telephone-CPR (t-CPR) instructions to callers has been shown to decrease time to first chest compression and to improve patient survival rate [1]. A t-CPR protocol has been introduced and routinely followed in Saudi Arabian emergency medical services (EMS) since 2017. However, callers first chest compression and patients return of spontaneous circulation (ROSC) have not been investigated yet. This study aims to examine callers first chest compression time and patients ROSC rate.

**Method**

A retrospective observational analysis of OHCA calls made to EMS in Riyadh, Saudi Arabia was conducted. OHCA patients were identified by reviewing archived EMS patients reports, then calls reported for these patients were retrieved from the EMS calling system. A sample of one hundred consecutive non-traumatic OHCAs of adult patients reported by adult Arabic spoken callers from January 2017-July 2018 was selected.

**Results**

Three hundred and eight confirmed OHCAs were identified and 100 were included. The median time from call receipt to start first chest compression (N=56) was 367 seconds (P=.004) with 267-551 interquartile range. Only four callers (8%) performed chest compression within the recommended AHA standard. ROSC was 10% on hospital arrival.

**Conclusion**

There is a significant delay in time to start first chest compression with a median time more than double the AHA standard, which might have affected the ROSC rate. Further investigation to examine the t-CPR protocol to identify areas for improvement with particular focus on time to first chest compression is recommended.

**References**

1. Perkins GD, Handley AJ, Koster RW, Castren M, Smyth MA, Olasveengen T, et al. European Resuscitation Council Guidelines for Resuscitation 2015: Section 2. Adult basic life support and automated external defibrillation. Resuscitation. 2015;95:81-99.

2. American Heart Association Database [https://cpr.heart.org/en/resuscitation-science/telephone-cpr/t-cpr-recommendations-and-performance-measures] Accessed 05/07/2018

## 54. Incidence of Hyperoxia in HEMS Trauma Patients

### Patricia Leitch, Anthony Hudson

#### Emergency Department, St. Georges University Hospital, London, UK

##### **Correspondence:** Patricia Leitch (Patricia.leitch@nhs.net)

**Background**

Rapid sequence induction (RSI) is the gold-standard for prehospital airway protection. Following RSI trauma patients are typically ventilated using an inspired oxygen fraction (FiO2) of 1.0, irrespective of the presence or absence of hypoxaemia. High quality evidence associates hyperoxia with increased risk of morbidity and mortality in various subsets of critically ill patients, including major trauma [1].

**Method**

A retrospective review of the Air Ambulance Kent Surrey Sussex (AAKSS) database (HEMSbase) was undertaken to identify intubated trauma patients transferred to St George’s Hospital between October 2014 and May 2019. Pre-hospital data collected included: RSI time, FiO2 and pulse oximetry saturations (SpO2) on arrival. In-hospital data included the first arterial blood gas (ABG) taken on arrival. ABGs taken >2 hours following admission were excluded. The cohort was categorised into four oxygen exposure groups based on previous studies: hypoxia < 60 mmHg, normoxia 60–120 mmHg, mild hyperoxia 120–200 mmHg, and severe hyperoxia as >200 mmHg [2,3].

**Results**

209 patients were included in the final analysis. 59% (n = 124) were severely hyperoxic, 20% (n = 41) had mild hyperoxia, 19% (n = 40) were normoxic and 2% patients (n = 4) were hypoxic. Average arterial oxygen tension (PaO2) for the cohort was 268mmHg, maximal PaO2 recorded was 644mmHg and minimal was 50mmHg. Average time from RSI to arrival was 59 minutes. Arrival SpO2 was recorded in 200 patients, 75% (n = 150) had saturations >98% on arrival. Only 3 patients were documented as having their FiO2 reduced to 0.5 during the pre-hospital phase.

**Conclusion**

Hyperoxia exposure is common in the intubated HEMS trauma patient (79%). Prospective analysis is needed to define the incidence and impact of hyperoxia in the pre-hospital phase. In addition, strategies to avoid hyperoxia need to be developed to avoid unnecessary harm to our patients.

**References**

1. Chu, D., Kim, L., Young, P., Zamiri, N., Almenawer, S., Jaeschke, R., Szczeklik, W., Schünemann, H., Neary, J. and Alhazzani, W. (2018). Mortality and morbidity in acutely ill adults treated with liberal versus conservative oxygen therapy (IOTA): a systematic review and meta-analysis. The Lancet, 391(10131), pp.1693-1705.

2. Helmerhorst H, Arts D, Schultz M, van der Voort P, Abu-Hanna A, de Jonge E et al. Metrics of Arterial Hyperoxia and Associated Outcomes in Critical Care*. Critical Care Medicine. 2017;45(2):187-195.

3. Page D, Ablordeppey E, Wessman B, Mohr N, Trzeciak S, Kollef M et al. Emergency department hyperoxia is associated with increased mortality in mechanically ventilated patients: a cohort study. Critical Care. 2018;22(1).

## 55. Penetrating Gluteal Injuries in North West London

### Gerard McKnight^1^, Seema Yalamanchili^2^, Natalia Sanchez-Thompson^3^, Nadia Guidozzi^2^, Nicola Batrick^2^, Shehan Hettiaratchy^2^, Mansoor Khan^2^, Elika Kashef^2^, Chris Aylwin^2^, Dan Frith^2^

#### ^1^Institute of Naval Medicine, Royal Navy, UK; ^2^St Mary’s Hospital, London, UK; ^3^Charing Cross Hospital, London, UK

##### **Correspondence:** Gerard McKnight (gerard.mcknight@nhs.net)

**Background**

Penetrating gluteal injuries (PGI) are an increasingly common presentation to Major Trauma Centres (MTCs) in London [1]. PGI’s can be associated with significant morbidity, and a mortality of up to 10% [2]. This study analysed 2 years’ worth of data at St Mary’s MTC in North West London to assess the prevalence and to identify any trends that could be used to aid decision making in the Emergency Department.

**Methods**

Retrospective analysis of electronic health records of all patients presenting with a PGI to St Mary’s MTC over a 2 year period during the recent period of rising knife crime.

**Results**

There were 106 presentations in this period, 95.4% were male with a median age of 21 and a significant minority of under 18’s (22.6%). There were no sciatic nerve injuries but there were 4 rectal injuries (3.63%). Significantly, the patients stabbed in the lower inner quadrant of the buttock had an absolute risk of rectal injury of 16%. There were 16 vessel injuries that were spread evenly across the anatomical quadrants. The absolute risk of injury to either a vessel or bowel was 19%. There was also significant variation in the imaging techniques to assess these patients with an almost 50:50 split between dual phase CT and single phase CT.

**Conclusion**

PGI are a common presentation, representing 1.6% of all trauma calls or approximately one per week at our MTC, and have a significant risk of morbidity (19%). There is variation in imaging and further management. This study has demonstrated that the anatomical quadrant of injury is helpful in adjusting threshold of suspicion for rectal injury but not for assessing vessel injury. Based on this study we have developed a standardised PGI Treatment Pathway with multidisciplinary input for these patients at our MTC.

**References**

1. Campion T, Cross S. The spectrum of injuries in buttock stab wounds. Clinical Radiology 2017; 72: 543-551

2. Clemens M, Peace K, Yi F. Rectal Trauma: Evidence-Based Practices. Clinics in Colon and Rectal Surgery 2018; 31(1):17-23.

## 56. Blunt thoracotomy practice and outcomes in North West London

### Samir Abdelkarim, Seema Yalamanchili, Jasmine Winter-Beatty, Joanne Graves & Christopher Aylwin

#### St Mary’s Hospital, London, UK

##### **Correspondence:** Samir Abdelkarim (samir.abdelkarim@nhs.net)

**Background**

Resuscitative thoracotomy is performed for both blunt and penetrating injury, though the precise indications for such intervention in blunt trauma are debated. Evidence for thoracotomy for blunt trauma is limited and there is variation in protocol in the United Kingdom and internationally [1]. St Mary’s Hospital is a busy Major Trauma Centre serving North West London, which sees over 3,000 trauma patients annually. We reviewed presentations of blunt injury where thoracotomy was performed, to evaluate how our experience compares to the published literature and identify areas for change in practice.

**Methods**

We retrospectively reviewed blunt trauma cases from April 2015 and April 2019 using a combination of local and TARN databases and deeper exploration of electronic health records. Cases were reviewed for demographics, injury characteristics, location thoracotomy was performed (pre-hospital, emergency department or theatre), cardiac arrest, neurological outcome and survival.

**Results**

We identified 15 cases of resuscitative thoracotomy for blunt trauma, aged 12-83. Mechanisms of injury were RTCs (7), falls greater than 2 metres (6), fall from standing (1) and fall under train (1). 4 cases underwent thoracotomy pre-hospital, 3 in the emergency department and 8 in theatre. The 5 survivors (aged 30-83) all had thoracotomies in theatre and no subsequent neurological deficits. 4 had predominantly intrathoracic injuries, but 1 had intra- and extra-thoracic injuries.

**Conclusions**

Our experience demonstrates the utility of resuscitative thoracotomy for blunt trauma in selected cases. Notably, these surviving patients had not experienced circulatory arrest prior to reaching theatre, nor did they have a heavy burden of injury (as defined by ISS). We did not identify any surviving cases from the field nor the emergency department. Furthermore, this series also demonstrated survival in elderly patients who met these criteria, suggesting age alone may not be a negative predictive factor for positive outcomes.

**Reference**

1. Slessor D, Hunter S. To be blunt: are we wasting our time? Emergency department thoracotomy following blunt trauma: a systematic review and meta-analysis. Ann Emerg Med. 2015 Mar; 65(3):297-307.e16.

## 57. Do elderly trauma patients get a poor deal in a busy London MTC?

### Sarah Aspland^1^, Anne Weaver^2^

#### ^1^Medical Student, BSc Urgent and Emergency Care Plymouth University, The Royal London Hospital, London UK; ^2^Consultant in Emergency Medicine and Pre-Hospital Care, Clinical Director for Trauma, The Royal London Hospital, London UK

##### **Correspondence:** Sarah Aspland (sarah.aspland@nhs.net)

**Background**

London Major Trauma Centres are renowned for receiving a high proportion of penetrating trauma, activating ambulance service pre-alert and trauma team response almost without fail. Conversely, elderly patients with low energy mechanisms of injury are more likely to arrive without a pre-alert and be processed in a similar way to “non-trauma patients”. Does the lack of trauma call adversely affect outcome in our elderly patients?

**Methods**

A retrospective review of 50 patient records was performed incorporating MTC clinical notes, imaging reports and TARN submission forms. Inclusion criteria were age >65 years, presenting with traumatic injury and RLH Emergency Department attendance between 01/07/2018 and 01/01/2019. Key data collected included mechanism of injury, main injury sustained, time from arrival to CT scan, and pre-alert placement by ambulance service. 232 patients were identified, of which 50 were selected using an online randomiser.

**Results**

Fall from <2m was the predominant mechanism (72%) (*n=*36), with 52% (*n=*26) sustaining an ISS >15. Head injury was most common at 38% (*n=*18). 66% (*n=*33) of patients found to have a “significant” traumatic injury were not pre-alerted by the ambulance service, and 33% (*n=*17) of all patients did not trigger a trauma team response. The median time to CT scan from arrival was 1.05 hours. 12% of patients studied (*n=*6) died as a direct result of their injuries.

**Conclusion**

Our findings suggest that a high proportion of elderly patients with a low energy mechanism suffer significant injury which may not be apparent to prehospital or ED triage teams. A bespoke elderly trauma triage tool could provide decision support as to which patients may benefit from Trauma Team activation. Our audit suggests that non-trauma called patients have extended times to CT which could prove detrimental. Future work is required to clarify whether triage tools can improve key performance indicators and clinical outcomes.

## 58. Primary Percutaneous Coronary Intervention (pPCI) after Out of Hospital Cardiac Arrest (OHCA)

### Anna Essl^1,2^, Michael Eichlseder^1^, Laurie Phillipson^3^

#### ^1^Medizinercorps Graz Austrian Red Cross, Graz, Styria, Austria; ^2^LKH Hochsteiermark Standort Bruck an der Mur, Styria, Austria; ^3^Essex and Herts Air Ambulance, Earls Colne, Essex, UK

##### **Correspondence:** Anna Essl (anna.essl@gmail.com)

**Background**

The most common cause of OHCA is cardiac disease and sudden cardiac death is often the first manifestation of cardiac disease.^1^ Close partnerships with PCI centres are important for on-scene communication and joint triage decision-making. This work provides an overview of ROSC patients with cardiac disease and possible pPCI.

**Methods**

Using the EHAATs documentation platform, HEMSbase 2.0, a retrospective data research was performed. All conveyed OHCA with suspected cardiac cause between 05/18 and 10/19 were included. The only exclusion criteria were respiratory, metabolic or toxicological causes of OHCA incorrectly coded in HEMSbase.

**Results**

237 patients were investigated. 15% got excluded. In 30% no PCI center was called. From the remaining 131 patients 79% got accepted for pPCI. In general, accepted patients were younger (median age 58,7 vs. 63,7 years), had shorter low flow times (median time 27,4 vs. 35,1 min), recieved less adrenaline (median dose 1,7 vs. 2,8 mg), had a higher GCS on recovery (median GCS 5,7 vs 4,4 points) and an initial shockable rhythm was more common (89% vs 56%). 94% of accepted patients had witnessed arrest, 88% got bystander CPR, had less CV risk factors and less comorbidities (51 vs 78% and 50 vs 74%). 65% of accepted patients had STEMI but also patients with NSTEMI and ongoing CPR got accepted. 22% of not accepted patients had STEMI and 37% had NSTEMI. Regarding the hospitals Barts (100%), Harefield (100%), Papworth (92%), and Basildon (77%) had the highest accepting rates.

**Conclusion**

Acceptance ratios vary between PCI centres but known favourable prognostic factors seem to match acceptance criteria. For 30% of patients with assumed cardiac cause of OHCA, PCI centres were not contacted. 22% (n=6) of STEMI patients were not accepted directly for pPCI. 37% (n=10) of high- risk NSTEMI patients were not accepted directly for pPCI.

^1^ Jacob C. et al. 2019. Cardiopulmonary Resuscitation and Critical Care after Cardiac Arrest, Cardiac Intensive Care (Third Edition).

## 59. A comparison of assault-related and self-inflicted stab injuries in a rural HEMS service

### Michael Eichlseder^1^, Anna Essl^1^, Laurie Phillipson^2^

#### ^1^Medizinercorps Graz Austrian Red Cross, Graz, Styria, Austria; ^2^Essex and Herts Air Ambulance, Earls Colne, Essex, UK

##### **Correspondence:** Michael Eichlseder (Michael.eichlseder@st.roteskreuz.at)

**Background**

Since 2014, the number of knife crime offences in England and Wales has almost doubled [1]. Suicides and self-harm are increasing as well (12% from 2017 to 2018) [2]. We therefore aim to compare the data on assault-related and self-inflicted stab injuries attended by EHAAT.

**Methods**

Using the EHAATs documentation platform, HEMSbase 2.0, a retrospective data analysis was performed. Cases between December 1, 2014 and October 15, 2019 were searched for “self-inflicted injury by sharp object” and “assault by sharp object”. This resulted in 63 cases for the self-inflicted group (group 1) and 197 cases for the assault group (group 2). 21 had to be excluded from group 1 and 19 from group 2. Cases were searched for age, gender, location of injury and interventions.

**Results**

The median age in group 1 is 47 and 27 in group 2. In group 1, 60% of the patients were males, while in group 2 it was 94%. Patients in group 2 required more interventions from the HEMS team, for example a thoracotomy was performed in 8.43% versus 0% and a special haemostatic intervention (tourniquet, suture, haemostatic gauze) in 14.04% versus 4.76%. The location of injury varied as well, with group 1 patients having more neck (28.57% versus 12.36%) and abdominal (45.24% versus 36.52%) and group 2 having more thoracic (55.62% versus 40.48%) and upper leg (23.03% versus 2.38%) injuries.

**Conclusion**

The population in the assault group is younger and almost exclusively males. They have a higher risk of bleeding and need more interventions, as they are more often injured on their proximal extremeties and thorax. The incidents in the self-inflicted group are almost exclusively on the neck, thorax, abdomen and the lower arms.

Both injury mechanisms carry a high risk of significant morbidity and mortality. Public health and prevention strategies should be focussed on the concerning rise in incidents.

1. Allen G, Audickas G, Loft P, Bellis A. Knife Crime in England and Wales. House of Commons Library. 2019

2. Simms C, Scowcroft E, Isaksen M, Potter J, Morrissey J. Suicide statistics report, Latest statistics for the UK and Republic of Ireland. 2019

